# Starlng: STability Analysis of coRegulated ‘Nests’ of Genes

**DOI:** 10.1093/bioinformatics/btag801

**Published:** 2026-10-28

**Authors:** Andi Munteanu

**Affiliations:** Faculty of Computer Science, Alexandru Ioan Cuza University, Iași, 700483, Romania; Cambridge Stem Cell Institute, University of Cambridge, Cambridge, CB2 0AW, United Kingdom

## Abstract

**Motivation:**

Single-cell assays (both single and multi-omics) represent the state-of-the-art for detailed characterization of cell phenotypes, a task frequently initialized with the identification of groups of genes linked to well-defined, stable subpopulations of cells. Gene expression covariation patterns are simplified precursors of Gene Regulatory Networks (GRNs) and play a crucial role in inferring causality and dynamics within GRNs. However, current gene clustering methods struggle to capture spatial organization and relationship dynamics along developmental trajectories, while remaining vulnerable to noise and algorithmic stochasticity.

**Results:**

Expanding on the ClustAssess framework, which shifted the cluster-centric assessment of populations of cells to an element-centric perspective, we propose to assess the covariation induced by gene graph clustering. The Starlng (abbrev) framework provides an optimized, interactive environment for the identification of the filtered modules. The pipeline identifies the stable configuration of clustering parameters and robust gene modules (nests of genes), followed by the removal of the outlier clusters based on the pseudotime variance. We illustrate Starlng on the identification of reliable, reproducible communities of genes, as well as reveal unbiased hierarchical relationships between higher level cell clusters and more granular subgroups of genes.

**Availability and implementation:**

*Starlng* is implemented in R and freely available at https://github.com/Core-Bioinformatics/Starlng.

## 1 Introduction

The rapid development of single-cell technologies across modalities led to significant breakthroughs, both in terms of sequencing outputs and dynamic ranges, and in the number of samples per experiment, thus expanding the scope of biological studies (Regev *et al.* 2017). These advancements present ongoing challenges in identifying heterogeneous cell subpopulations, a crucial step for characterizing the transitions between different cell types (Gribben *et al.* 2024) or for understanding cell fate decisions and dynamic cellular processes (Trapnell 2015).

A focus of single-cell methods is linking gene expression patterns to observed phenotypes. The transition from manual curation to the use of automated, computational approaches promoted significant discoveries that expanded our understanding of gene functional mechanisms (Kiselev *et al*. 2019). These approaches rely on various techniques that range from extrapolating a manually annotated database (Pliner *et al*. 2019, Stock *et al.* 2025) of markers to statistical models and machine learning algorithms that focus on correlation and co-expression patterns of genes (Langfelder and Horvath 2008, Morabito *et al.* 2023).

The assessment of gene-centric pairwise covariation patterns enabled the evaluation of regulatory interactions, leading to an incremental construction of comprehensive gene regulatory networks (GRNs) at the genome-wide level (Huynh-Thu *et al.* 2010, Aibar *et al.* 2017). Recent studies reinforce this, showing how GRNs successfully capture behavioural patterns globally and locally (Hartl *et al.* 2021).

Current research efforts aim to enhance the granularity of GRN inference methods, which were traditionally applied to bulk transcriptomics data (Langfelder and Horvath 2008). There is growing interest in improving the specificity of local networks (Cui *et al.* 2022, Jia *et al.* 2023), identifying key regulatory hubs (Xu *et al.* 2022, Gao *et al.* 2024) and uncovering dynamic interaction patterns across multiple developmental stages (Song and Li 2021, Song *et al*. 2022). The computational approaches addressing this task span a wide spectrum of algorithms, from statistical models to machine learning-inspired methods, all with an emphasis on flexibility in defining local network structures and enabling the exploration of hierarchical relationships within regulatory systems (Langfelder *et al*. 2008).

A significant challenge in GRN inference is represented by the robustness and reproducibility of the results (Lähnemann *et al.* 2020). Benchmark studies on real-world single-cell datasets revealed substantial variability in network inference outcomes across methods and datasets, underscoring the need for frameworks that assess and ensure the stability of GRN results (Langfelder *et al.* 2011, Saelens *et al*. 2018, Kang *et al*. 2021, Chevalley *et al.* 2025). Addressing the bias in choosing the target group of genes (Aibar *et al.* 2017) would be a step forward in ensuring the robustness of the results.

To bridge stability analyses and covariation patterns inference, and ultimately GRNs, we introduce Starlng (STability Analysis of coRreguLated Nests of Genes), a framework designed to evaluate and improve the robustness of graph-based gene clustering, leading to the identification of gene modules that characterize transitions between cell states across pseudotime trajectories. Starlng leverages the stability assessment principles from the ClustAssess framework (Shahsavari *et al*. 2022) to find optimal configurations of parameters and gene modules, highly reproducible across multiple runs. Starlng determines the quality of the resulting modules and automatically discards the outliers. The filtered clusters are then used to define and functionally characterize the subpopulation that drives the transition.

## 2 Materials and methods

Starlng was applied to the immune cells from a MASLD/MASH study (Gribben *et al.* 2024) and to the Liver Hepatoblasts and Pancreas Acinar, Ductal and Islet endocrine cells from the Cao *et al.* (2020) Human cell atlas. Quality checks and filters were aligned with the original manuscripts. The normalization and scaling of the count matrix was performed using the *SCTransform* function from Seurat v.5.1.0 (Hao *et al.* 2024). For the fetal cell atlas, we used *Log-Normalization*.

All experiments were performed on a high-memory machine (Dell Precision 7780, Intel i9-13950HX processor with 32 cores, 128 GB RAM, Ubuntu 22.04.4 LTS Linux distro used under WSL2). A detailed description of the software environment is provided in Section 9, available as supplementary data at *Bioinformatics* online.

## 3 Results

### 3.1 Starlng framework

Monocle3 (Cao *et al.* 2019) was one of the first frameworks which used the PhenoGraph pipeline (Levine *et al.* 2015) to determine the modules of coexpressed genes. Acknowledging the coexistence of signal and noise (Moutsopoulos *et al.* 2021), this approach was accompanied by a pre-filtering of the genes based on an autocorrelation metric. Monocle3 opted to apply Moran’s I test (Moran 1950 ) on the inferred trajectory graph.

We developed Starlng, an R framework for stability-aware trajectory-guided gene module discovery that extends Monocle3. The workflow is presented in Fig. 1a.

**Figure 1:**
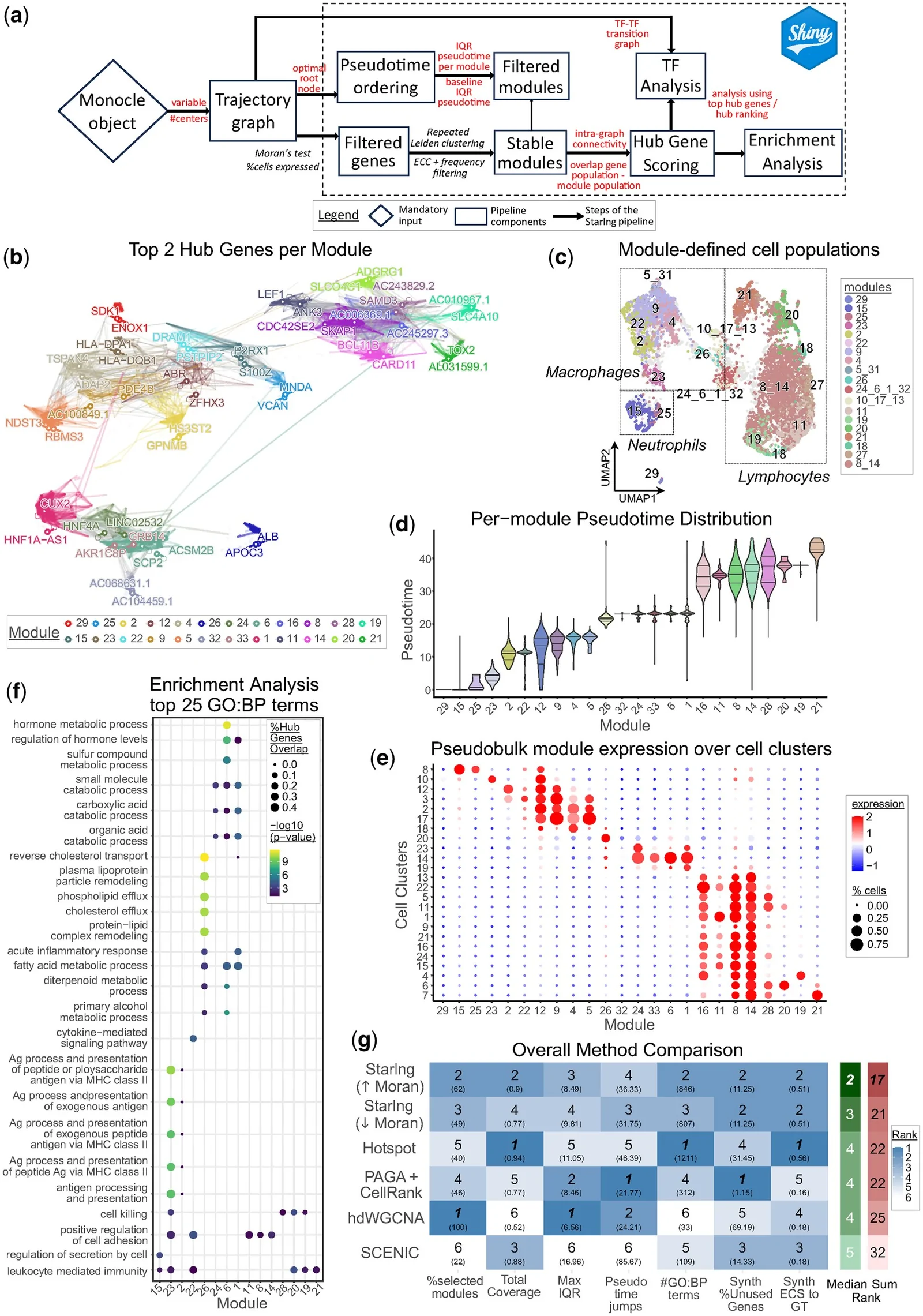
Starlng workflow and results. (a) Workflow diagram showing the main steps of the Starlng framework. The red text describes main contributions from Starlng pipeline. The Shiny logo illustrates that, starting with pseudotime ordering and gene filtering, each component can be executed in real time in a Shiny environment. (b) 2D representation of the genes, coloured by the modules identified and filtered by Starlng. The UMAP was built on the feature loading calculated using PCA at the cell level. For each module, the names of its top 2 hub genes are displayed. Top 25% of the edges (ordered by the SNN weights) are shown. (c) 2D representation of the cell populations defined by the filtered Starlng gene modules. The cell UMAP was used to show the coverage of the inferred population across all three cell types. (d) Violin plot showing the distribution of pseudotime (*y*-axis) of the filtered Starlng gene modules (*x*-axis). Three main groups can be noticed. The plot also helps visualize the tightness of the distribution, as well as the transitioning points (such as modules 23, 2, 22). (e) Bubbleplot showing the association between the stable 24 cell clusters identified by ClustAssess and the filtered gene modules. The size of the point is proportional to the number of cells from the clusters that are identified by a module. The colour gradient indicates the strength of the scaled average expression of the genes belonging to the module. (f) Bubbleplot showing the modules that were used to identify the top 25 enriched GO: BP terms. The size of the point is proportional to the percentage of top 15 hub genes that are used to identify the term. The colour gradient reflects the *p*-value significance (bright colour–low *p*-value). (g) Table showing the results of the overall comparison between Starlng and the other four gene clustering frameworks (the arrows indicate whether the Moran’s threshold is high or not). The column are associated to the following metrics: percentage of selected modules, total module coverage, highest pseudotime IQR of a module, the sum of pseudotime jumps, percentage of genes not clustered in the SERGIO datasets, the ECS score between the gene modules and the SERGIO reference clusters. Each cell contains the ranking that a method achieved for a particular metric, the actual value being mentioned in the parentheses. The median and sum rank dictate the overall ranking of the method. Starlng demonstrates the best performance on this dataset.

Firstly, Starlng calculates the trajectory graph using an extended version of the *learn_graph* function from Monocle3 that provides better control on the parameters, i.e. the ratio between the graph nodes and the size of the data. This is immediately followed by the inference of the pseudotime ordering. For this step, Starlng automatically identifies the starting point that maximizes the pseudotime range.

Afterwards, the autocorrelation score is calculated and then used to filter out genes that do not show population-specific patterns. The remaining features are then clustered using the Leiden community detection algorithm (Traag *et al*. 2019, Ewing 2026). Starlng builds on the ClustAssess framework (Shahsavari *et al*. 2022) and selects the number of clusters that show high Element-Centric Consistency (ECC) across multiple runs.

The resulting stable modules are further filtered based on their consistency with associated cell populations, as quantified by pseudotime dispersion and spatial coherence in UMAP space. Starlng introduces an intermediate labeling scheme to identify redundant modules, defined as those whose associated populations span broad regions of pseudotime and substantially overlap with populations already captured by more specific gene clusters.

For each module, we employ a composite scoring metric that quantifies the importance of each gene based on two complementary signals: its intra-modular interaction within the gene similarity graph and its overlap with the module’s aggregate expression profile.

Finally, the results are incorporated into a single interactive Shiny web application (Chang *et al*. 2026) that expands the analysis of the modules and their functions. Detailed summaries and method descriptions are provided in Sections 1 and 2, available as supplementary data at *Bioinformatics* online.

### 3.2 Applying Starlng on experimental data

We exemplify the Starlng framework on the immune subset of the Gribben *et al.* (2024) MASLD/MASH dataset. The Starlng identifies the 40-Shared Nearest Neighbour graph (Csárdi *et al*. 2026, Jefferis *et al*. 2026) as the optimal configuration in terms of stability (Fig. 1a, available as supplementary data at *Bioinformatics* online). The available number of modules ranges between 4 and 38. Figure 1b, available as supplementary data at *Bioinformatics* online shows the stability of each value, defined by the average ECC score and frequency. The highest number of stable modules, 37, was selected. The outlier and redundant partitions were excluded from the analysis, leaving 24 usable modules. We describe the impact of the module filtering in Section 3, available as supplementary data at *Bioinformatics* online.

Projecting the filtered modules on a 2D UMAP space (McInnes *et al*. 2018)  shows a clear grouping in three main partitions (Fig. 1b). The violin plot from Fig. 1d contains the pseudotime distribution of the cells that are defined by each gene module. It confirms the presence of three main partitions: from module 29 to 5, 26 to 1, and 16 to 21.

To understand the identity of module-defined populations, we display their unified distribution on the reduced space of the cells. Figure 1c shows that all three main cell types are well represented by more than one module. This plot also helps us map the relationship between the three main partitions and cell annotations. This mapping is quantified in Fig. 1e via pseudobulk association between cell clusters and the modules. Notably, every ClustAsssess cluster is described by at least one module.

Analysing panels C and D together drive the conclusion that the modules display good complementarity in describing a particular transitioning state across the pseudotime ordering. Figure 2, available as supplementary data at *Bioinformatics* online details the specific population associated with each module.

To functionally interpret the filtered modules, we performed enrichment analysis (Kolberg *et al*. 2020)  against GO: BP (Ashburner *et al.* 2000; top 25 terms shown in Fig. 1f) and TRANSFAC (Matys *et al.* 2006) databases (top 10 terms shown in Fig. 3a, available as supplementary data at *Bioinformatics* online). Several established markers from the Gribben *et al.* (2024) manuscript are recapitulated among the top 15 hub genes: *CD247, GNLY, CD96, BCL11B* for lymphocytes; *GAS7, MYO9A* for macrophages; and *FCN1, MNDA, VCAN-AS1, VCAN* for neutrophils. Starlng identifies relevant biological processes for all present cell types. For example, lymphocyte-associated module 28 was enriched for “cell killing” and “leukocyte-mediated immunity processes” (Martin *et al.* 2025), in conjunction with transcription factors such as *LEF-1, TCF-4*, and *beta-catenin* (Liu *et al.* 2022). Macrophage-associated module 26 was enriched for terms such as fatty acid metabolic process and reverse cholesterol transport that are linked to the function of lipid-associated macrophages (Jaitin *et al.* 2019). This interpretation is further supported by the enrichment of the *HNF4-alpha* transcription factor motif, consistent with its established role in hepatocyte metabolic homeostasis and liver-specific gene regulation (Radi *et al.* 2023).

To evaluate the robustness, we successfully applied the Starlng algorithm to two additional datasets. The results are presented in Section 4, available as supplementary data at *Bioinformatics* online.

### 3.3 Comparison with other methods

In the previous sections, we presented the workflow of Starlng and its ability to capture the biological identity of the cell populations. In this section, we benchmark Starlng against four state-of-the-art methods: SCENIC (Aibar *et al.* 2017), hdWGCNA (Morabito *et al.* 2023), PAGA + CellRank (Wolf *et al.* 2019, Lange *et al.* 2022), and Hotspot (DeTomaso and Yosef 2021). Detailed benchmarking and comparison methodologies are provided in Section 1, available as supplementary data at *Bioinformatics* online.

First, we evaluate these methods on a real dataset using the MASLD/MASH Immune dataset introduced previously. In this comparison, we introduced two runs of Starlng: one with the default filtering (Moran’s *I* > 0.1) and one with a more permissive threshold (Moran’s *I* > 0.01). A direct comparison between these two operational thresholds is provided in Section 5, available as supplementary data at *Bioinformatics* online.

Figure 4, available as supplementary data at *Bioinformatics* online visually highlights stark differences in cluster numbers, pseudotime coverage, and signal-to-noise ratios across the workflows, using cell-resolution line plots (Wickham 2016) and heatmaps (Gu *et al*. 2016). The middle panel from Fig. 5a, available as supplementary data at *Bioinformatics* online shows that Starlng retains over 60% of the initial set of gene clusters. While hdWGCNA retained 100%, it identified only three modules compared to Starlng’s 37. Starlng achieved 90% cell coverage using the filtered modules (Fig. 5b, available as supplementary data at *Bioinformatics* online, middle panel). Hotspot provides marginally higher coverage (94%) but introduces modules with greater variance across pseudotime, yielding a maximum IQR of 11.05, compared to 8.49 for Starlng.

Starlng ranked third in trajectory continuity (Fig. 6b, available as supplementary data at *Bioinformatics* online), heavily impacted by a single gap between modules 33 and 16 (Fig. 6a, available as supplementary data at *Bioinformatics* online). In functional enrichment analysis (GO: BP and TRANSFAC) using the top 10 hub genes per module, Starlng achieved the second-highest number of enriched terms behind Hotspot (Fig. 5c, available as supplementary data at *Bioinformatics* online).

Next, we evaluated the methods against synthetic datasets with predefined regulatory interactions. Using the GRN inferred by SCENIC on the MASLD/MASH data, we utilized SERGIO (Dibaeinia and Sinha 2020) to generate 16 datasets varying in the number of gene modules, the presence of a known trajectory, and technical noise (UMAP visualization in Fig. 7, available as supplementary data at *Bioinformatics* online). Based on the Element-Centric Similarity (Gates *et al.* 2019) to the simulated reference clustering, Starlng achieves an average score above 0.8 on denoized counts (Fig. 8a, available as supplementary data at *Bioinformatics* online). The score drastically decreases when noise is added, but we remark that all methods are affected and perform worse than Starlng.

We have also compared the frameworks based on the percentage of genes that are retained for the analysis. Starlng achieves the second-best performance by discarding 11% of genes after Moran’s I filtering, compared to 69% in hdWGCNA and 1.15% in PAGA + CellRank (Fig. 8b, available as supplementary data at *Bioinformatics* online).

Finally, we ranked each method across all evaluation criteria. The cumulative ranking overview (Fig. 1g) demonstrates superior overall performance for Starlng, a conclusion consistently recapitulated across other datasets (Section 4, available as supplementary data at *Bioinformatics* online).

## 4 Discussion

Our findings highlight the need to define quality metrics that decide whether a gene module can be used in the analysis or not. Cluster filtering has improved the separation of module partitions. The results indicate that the filtered modules can be confidently used to describe any cell type in terms of coverage and functional interpretation. With the development of the Starlng Shiny app, we have also addressed the challenge of mediating data interrogation towards any user, irrespective of their informatics experience.

Starlng identifies trajectory-guided gene modules that capture gene-expression patterns across overlapping cell populations that may not align with discrete clusters. This provides a complementary perspective to conventional approaches that derive gene sets from cell clusters (as discussed in Section 6, available as supplementary data at *Bioinformatics* online).

Using the ClustAssess approach to improve the stability ensures robustness and reproducibility of the results. We evaluated the robustness of Starlng by comparing its results after subsampling, the introduction of noise and repeated runs with changing random seeds (detailed in Section 7, available as supplementary data at *Bioinformatics* online). Finally, we analysed the scalability of the framework. Our benchmarks demonstrate that runtime scales linearly with dataset size (as shown in Section 8, available as supplementary data at *Bioinformatics* online; Risso *et al*. 2018).

Further steps in the development of this idea would be linked to assessing both the stability and the quality of the gene modules in relationship with the pseudotime ordering and to the improvement of the relationship between the localized gene expression profiles and the inferred trajectory. Extending the flexibility towards other methods of pseudotime trajectory inference (Saelens *et al.* 2019) would also benefit the generalizability of the package.

## Supplementary Material

btag801_Supplementary_Data

## Data Availability

The Starlng package is publicly available as open-source on GitHub: https://github.com/Core-Bioinformatics/Starlng and is accessible via Zenodo at 10.5281/zenodo.20821317. The source code for the analysis from this manuscript is publicly available as open-source on GitHub: https://github.com/Core-Bioinformatics/Starlng_paper and is accessible via Zenodo at 10.5281/zenodo.23200421. The Starlng ShinyApps of the datasets analysed in this manuscript can be accessed at the following link: https://mohorianulab.org/shiny/Starlng/.

## References

[btag801-B1] Aibar S , González-Blas CB, Moerman T et al SCENIC: single-cell regulatory network inference and clustering. Nat Methods 2017;14:1083–6. 10.1038/nmeth.4463 PMC5937676 28991892

[btag801-B4] Ashburner M , Ball CA, Blake JA et al Gene ontology: tool for the unification of biology. Nat Genet 2000;25:25–9. 10.1038/75556 PMC3037419 10802651

[btag801-B6] Cao J , O’Day DR, Pliner HA et al A human cell atlas of fetal gene expression. Science 2020;370:eaba7721. 10.1126/science.aba7721 PMC7780123 33184181

[btag801-B7] Cao J , Spielmann M, Qiu X et al The single-cell transcriptional landscape of mammalian organogenesis. Nature 2019;566:496–502. 10.1038/s41586-019-0969-x PMC6434952 30787437

[btag801-B9] Chang W, Cheng J, Allaire JJ et al Shiny: web application framework for R. Version 1.14.0. 21 June 2026.

[btag801-B10] Chevalley M , Roohani YH, Mehrjou A et al A large-scale benchmark for network inference from single-cell perturbation data. Commun Biol 2025;8:412. 10.1038/s42003-025-07764-y PMC11897147 40069299

[btag801-B13] Csárdi G, Nepusz T, Müller K et al igraph for R: R interface of the igraph library for graph theory and network analysis. 17 July 2026.

[btag801-B14] Cui EH , Song D, Wong WK et al Single-cell generalized trend model (scGTM): a flexible and interpretable model of gene expression trend along cell pseudotime. Bioinformatics 2022;38:3927–34. 10.1093/bioinformatics/btac423 PMC9991897 35758616

[btag801-B15] DeTomaso D , Yosef N. Hotspot identifies informative gene modules across modalities of single-cell genomics. Cell Syst 2021;12:446–56.e9. 10.1016/j.cels.2021.04.005 33951459

[btag801-B16] Dibaeinia P , Sinha S. SERGIO: a single-cell expression simulator guided by gene regulatory networks. Cell Syst 2020;11:252–71.e11. 10.1016/j.cels.2020.08.003 PMC7530147 32871105

[btag801-B17] Ewing B, Traag V, Csárdi G et al Leidenbase: R and C/C++ wrappers to run the Leiden Find_partition() function. Version no. 0.1.37. 19 May 2026.

[btag801-B19] Gao Q , Ji Z, Wang L et al SifiNet: a robust and accurate method to identify feature gene sets and annotate cells. Nucleic Acids Res 2024;52:e46. 10.1093/nar/gkae307 PMC11109959 38647069

[btag801-B20] Gates AJ , Wood IB, Hetrick WP et al Element-centric clustering comparison unifies overlaps and hierarchy. Sci Rep 2019;9:8574. 10.1038/s41598-019-44892-y PMC6561975 31189888

[btag801-B22] Gribben C , Galanakis V, Calderwood A et al Acquisition of epithelial plasticity in human chronic liver disease. Nature 2024;630:166–73. 10.1038/s41586-024-07465-2 PMC11153150 38778114

[btag801-B23] Gu Z , Eils R, Schlesner M. Complex heatmaps reveal patterns and correlations in multidimensional genomic data. Bioinformatics 2016;32:2847–9. 10.1093/bioinformatics/btw313 27207943

[btag801-B25] Hao Y , Stuart T, Kowalski MH et al Dictionary learning for integrative, multimodal and scalable single-cell analysis. Nat Biotechnol 2024;42:293–304. 10.1038/s41587-023-01767-y PMC10928517 37231261

[btag801-B26] Hartl CL , Ramaswami G, Pembroke WG et al Coexpression network architecture reveals the brain-wide and multiregional basis of disease susceptibility. Nat Neurosci 2021;24:1313–23. 10.1038/s41593-021-00887-5 PMC10263365 34294919

[btag801-B27] Huynh-Thu V , Irrthum A, Wehenkel L et al Inferring regulatory networks from expression data using tree-based methods. Plos One 2010;5:e12776. 10.1371/journal.pone.0012776 PMC2946910 20927193

[btag801-B28] Jaitin DA , Adlung L, Thaiss CA et al Lipid-associated macrophages control metabolic homeostasis in a Trem2-Dependent manner. Cell 2019;178:686–98.e14. 10.1016/j.cell.2019.05.054 PMC7068689 31257031

[btag801-B29] Jefferis G , Kemp SE, Arya S et al RANN: fast nearest neighbour search (Wraps ANN Library) Using L2 Metric. Version 2.6.3. 24 August 2026.

[btag801-B30] Jia Y , Wu H, Ding J. scGeneRythm: using neural networks and fourier transformation to cluster genes by time-frequency patterns in single-cell data. bioRxiv, 27 November 2023, preprint: not peer reviewed.

[btag801-B31] Kang Y , Thieffry D, Cantini L. Evaluating the reproducibility of single-cell gene regulatory network inference algorithms. Front Genet 2021;12:617282. 10.3389/fgene.2021.617282 PMC8019823 33828580

[btag801-B32] Kiselev VY , Andrews TS, Hemberg M. Challenges in unsupervised clustering of single-cell RNA-seq data. Nat Rev Genet 2019;20:273–82. 10.1038/s41576-018-0088-9 30617341

[btag801-B33] Kolberg L , Raudvere U, Kuzmin I et al gprofiler2—an R package for gene list functional enrichment analysis and namespace conversion toolset g: profiler. F1000Research 2020;9:ELIXIR-709. 10.12688/f1000research.24956.2 PMC7859841 33564394

[btag801-B34] Lähnemann D , Köster J, Szczurek E et al Eleven grand challenges in single-cell data science. Genome Biol 2020;21:31. 10.1186/s13059-020-1926-6 PMC7007675 32033589

[btag801-B35] Lange M , Bergen V, Klein M et al CellRank for directed single-cell fate mapping. Nat Methods 2022;19:159–70. 10.1038/s41592-021-01346-6 PMC8828480 35027767

[btag801-B36] Langfelder P , Horvath S. WGCNA: an R package for weighted correlation network analysis. BMC Bioinformatics 2008;9:559. 10.1186/1471-2105-9-559 PMC2631488 19114008

[btag801-B37] Langfelder P , Luo R, Oldham MC et al Is my network module preserved and reproducible? PLOS Comput Biol 2011;7:e1001057. 10.1371/journal.pcbi.1001057 PMC3024255 21283776

[btag801-B38] Langfelder P , Zhang B, Horvath S. Defining clusters from a hierarchical cluster tree: the dynamic tree cut package for R. Bioinformatics 2008;24:719–20. 10.1093/bioinformatics/btm563 18024473

[btag801-B40] Levine JH , Simonds EF, Bendall SC et al Data-driven phenotypic dissection of AML reveals progenitor-like cells that correlate with prognosis. Cell 2015;162:184–97. 10.1016/j.cell.2015.05.047 PMC4508757 26095251

[btag801-B41] Liu J , Xiao Q, Xiao J et al Wnt/β-catenin signalling: function, biological mechanisms, and therapeutic opportunities. Signal Transduct Target Ther 2022;7:3. 10.1038/s41392-021-00762-6 PMC8724284 34980884

[btag801-B42] Martin OP , Wallace MS, Oetheimer C et al Single-cell atlas of human liver and blood immune cells across fatty liver disease stages reveals distinct signatures linked to liver dysfunction and fibrogenesis. Nat Immunol 2025;26:1596–611. 10.1038/s41590-025-02255-y PMC12818073 40883571

[btag801-B43] Matys V , Kel-Margoulis OV, Fricke E et al TRANSFAC® and its module TRANSCompel®: transcriptional gene regulation in eukaryotes. Nucleic Acids Res 2006; 34:D108–10. 10.1093/nar/gkj143 PMC1347505 16381825

[btag801-B44] McInnes L , Healy J, Saul N et al UMAP: uniform manifold approximation and projection. JOSS 2018;3:861. 10.21105/joss.00861

[btag801-B45] Morabito S , Reese F, Rahimzadeh N et al hdWGCNA identifies co-expression networks in high-dimensional transcriptomics data. Cell Rep Methods 2023;3:100498. 10.1016/j.crmeth.2023.100498 PMC10326379 37426759

[btag801-B46] Moran PAP. A test for the serial independence of residuals. Biometrika 1950;37:178–81. 15420264

[btag801-B47] Moutsopoulos I , Maischak L, Lauzikaite E et al noisyR: enhancing biological signal in sequencing datasets by characterizing random technical noise. Nucleic Acids Res 2021;49:e83. 10.1093/nar/gkab433 PMC8373073 34076236

[btag801-B48] Pliner HA , Shendure J, Trapnell C. Supervised classification enables rapid annotation of cell atlases. Nat Methods 2019;16:983–6. 10.1038/s41592-019-0535-3 PMC6791524 31501545

[btag801-B49] Radi SH , Vemuri K, Martinez-Lomeli J et al HNF4α isoforms: the fraternal twin master regulators of liver function. Front Endocrinol (Lausanne) 2023;14:1226173. 10.3389/fendo.2023.1226173 PMC10438950 37600688

[btag801-B50] Regev A , Teichmann SA, Lander ES et al The human cell atlas. eLife 2017;6:e27041. 10.7554/eLife.27041 PMC5762154 29206104

[btag801-B52] Risso D , Perraudeau F, Gribkova S et al A general and flexible method for signal extraction from single-cell RNA-seq data. Nat Commun 2018;9:284. 10.1038/s41467-017-02554-5 PMC5773593 29348443

[btag801-B55] Saelens W , Cannoodt R, Saeys Y. A comprehensive evaluation of module detection methods for gene expression data. Nat Commun 2018;9:1090. 10.1038/s41467-018-03424-4 PMC5854612 29545622

[btag801-B56] Saelens W , Cannoodt R, Todorov H et al A comparison of single-cell trajectory inference methods. Nat Biotechnol 2019;37:547–54. 10.1038/s41587-019-0071-9 30936559

[btag801-B57] Shahsavari A , Munteanu A, Mohorianu I. ClustAssess: tools for assessing the robustness of single-cell clustering. bioRxiv, 2 February 2022, preprint: not peer reviewed.

[btag801-B58] Song D , Li JJ. PseudotimeDE: inference of differential gene expression along cell pseudotime with well-calibrated p-values from single-cell RNA sequencing data. Genome Biol 2021;22:124. 10.1186/s13059-021-02341-y PMC8082818 33926517

[btag801-B59] Song Q , Wang J, Bar-Joseph Z. scSTEM: clustering pseudotime ordered single-cell data. Genome Biol 2022;23:150. 10.1186/s13059-022-02716-9 PMC9264648 35799304

[btag801-B60] Stock M , Losert C, Zambon M et al Leveraging prior knowledge to infer gene regulatory networks from single-cell RNA-sequencing data. Mol Syst Biol 2025;21:214–30. 10.1038/s44320-025-00088-3 PMC11876610 39939367

[btag801-B63] Traag VA , Waltman L, Van Eck NJ. From Louvain to Leiden: guaranteeing well-connected communities. Sci Rep 2019;9:5233. 10.1038/s41598-019-41695-z PMC6435756 30914743

[btag801-B64] Trapnell C. Defining cell types and states with single-cell genomics. Genome Res 2015;25:1491–8. 10.1101/gr.190595.115 PMC4579334 26430159

[btag801-B65] Wickham H. ggplot2: Elegant Graphics for Data Analysis. Cham: Springer International Publishing, 2016.

[btag801-B66] Wolf FA , Hamey FK, Plass M et al PAGA: graph abstraction reconciles clustering with trajectory inference through a topology preserving map of single cells. Genome Biol 2019;20:59. 10.1186/s13059-019-1663-x PMC6425583 30890159

[btag801-B68] Xu Q , Li G, Osorio D et al scInTime: a computational method leveraging single-cell trajectory and gene regulatory networks to identify master regulators of cellular differentiation. Genes (Basel) 2022;13:371. 10.3390/genes13020371 PMC8872487 35205415

