# Supplementary material for: Starlng: STability Analysis of coRegulated ‘Nests’ of Genes
Source: Bioinformatics. 2026 Oct 28;42(11):btag801. doi: 10.1093/bioinformatics/btag801 (PMC13760217; doi:10.1093/bioinformatics/btag801)
Supplement: Supplementary file 1 [file btag801_supplementary_data.docx]

Supplement: Starlng: STability Analysis of coReguLated "Nests" of Genes

Andi Munteanu^1,2,*^

^1^Faculty of Computer Science, Alexandru Ioan Cuza University, Iași, Romania ^2^Cambridge Stem Cell Institute, University of Cambridge, CB2 0AW, UK.

*To whom correspondence should be addressed.

# Supplementary Information

## Section 1. Description of Methods

### Trajectory Graph Inference

PAGA (Wolf et al. 2019) suggested graph abstraction of the data by pseudobulking: each celltype is summarised in a super-node and the interaction between nodes is calculated based on the neighbourhood overlap. Monocle3 (Cao et al. 2019) uses this as a starting point to cluster the celltypes into partitions. For each partition, they apply the SimplePPT algorithm - given several centre nodes, their coordinates are determined as the centroids of the k-Means clusters. The MST is finally applied to identify the tree structure that connects the centroids.

The number of centre points impacts the granularity of the resulting trajectory graph. Monocle3 has employed a heuristic that states that each cluster should be represented by $k\cdot\log_{10} \left( N_{cells} \right)$ nodes. The algorithm has fixed the value of *k* to 15. In Starlng, we have addressed this and added a parameter that controls this adaptive factor. We analysed the impact of this parameter in Suppl. Section 10.

### Identification of Autocorrelated Genes

Spatial autocorrelation testing was introduced to single-cell trajectory analysis within the Monocle3 framework (Cao et al. 2019). The Moran’s I statistic (Moran 1950) was used to calculate the autocorrelation score for each gene by comparing its expression vector to the expression patterns of its neighbours. The neighbours are determined by applying the kNN algorithm on the UMAP reduced space in conjunction with the trajectory graph vicinity.

The values of Moran’s I score range between –1 and 1 and reflect the strength of the autocorrelation. Zero values are associated with a random spatial expression pattern, while higher positive values indicate a stronger clustering effect of the cells expressing the gene.

The authors of the Monocle3 framework suggested a filtering of the genes based on the significance of the statistical test (adjusted p value < 0.05). We introduced a second filtering step based on the raw Moran’s I value. A lenient threshold must exceed 0; for this, we proposed a threshold value of 0.01. We noticed in our experiments that increasing this threshold discards potentially noisy genes and improves the quality of the modules. To achieve this effect while maintaining a reasonable number of genes, we set the default value of the threshold to 0.1. We note, however, that the optimal value should be adapted based on the characteristics of the data.

Suppl. Section 6 provides an evaluation of the alignment between trajectory-guided autocorrelation, differential expression, and expression variability.

### Pseudotime Ordering

The inferred trajectory graph can be used to determine an ordering of the cells that would reflect various stages in the developmental process. Monocle3 introduces the pseudotime score. Each cell is assigned to the closest centroid with respect to the UMAP space. Then the pseudotime of the cell is calculated as the geodesic distance between the associated centroid and the root node.

Since the biological start of the trajectory is not known a priori, the selection of the root node remains an important challenge. The authors of Monocle3 proposed an approach of interactively choosing the starting point on the trajectory shown on UMAP. In Starlng, we expanded the available options by proposing multiple data-driven heuristics.

The first relies on existing categorical metadata. The intuition is to use categories (such as the gestational age) that can already provide a good ordering of the cell bulks. Selecting one or more categories leads to the calculation of the root node as the cell that is closest to the centroid median.

The second approach uses a voting scheme of one or multiple genes. This encourages defining the start of the trajectory using genes with literature-known mechanisms that are relevant to the data. For the voting scheme, the user can provide either a flat threshold that filters cells with low average expression, or an adaptive percentile that retains only the top percentage of expressing cells. Moreover, the function can reduce voting strictness by providing an acceptable margin (i.e. how many genes should be expressed simultaneously) for the number of voters. The output of the voting scheme is a population of cells that will be summarised using the geometric median. In both cases, the identification of the root node is enhanced by a prior filtering of outlier cells based on their distance to the median.

The third approach aims to maximise the pseudotime range. For this, we determine the diameter of the trajectory graph and select the pair of nodes with the longest shortest path as potential candidates. The choice will impact the direction of the ordering. Our approach is to identify the nodes from the main lineage where branching occurs and to calculate the length of the branch. Using the assumption of having a more unified starting point that tends to split across the developmental stage, we choose the candidate located on the diameter half with shorter branches. This result is confirmed and explained in Suppl. Section 10.

### Gene Clustering

For gene clustering, Monocle3 incorporates the PhenoGraph (Levine et al. 2015) community detection pipeline that is usually applied in the context of cell clustering (Cao et al. 2019; Hao et al. 2024). The pipeline comprises three steps: dimensionality reduction (linear, such as PCA or nonlinear, such as UMAP), graph construction, and graph clustering.

In the context of gene clustering, Monocle3 uses feature loading from the cell PCA as a substitute for the first step. For the second step, the kNN algorithm is applied to infer the neighbourhood of each gene. The weight of a link between two genes is determined by the Jaccard Similarity Index (JSI) of their neighbourhoods. An additional threshold removes weak links that do not impact the connectivity of the graph.

Finally, the Leiden (Traag, Waltman, Van Eck 2019) algorithm is used to identify gene communities (also named modules). The resolution parameter plays a significant role in the resulting number of clusters.

For our experiments, we varied the number of nearest neighbours between five and 50. To calculate the modularity in the context of community detection, we used Reichardt and Bornholdt’s Potts model (Reichardt, Bornholdt 2006). The resolution parameter was varied between 0.1 and five.

### Stability Assessment

The stochasticity of the algorithms does not ensure a deterministic result. The output can vary if the computational environment or the initial random state is changed. To address this, we applied the stability assessment principles from ClustAssess (Shahsavari, Munteanu, Mohorianu 2022) in the context of gene clustering.

The parameters we vary are the number of nearest neighbours (a default range of five to 50) and the resolution (a default range of 0.1 - two). We run the clustering pipeline multiple times with the same configuration, changing only the initial random state. By default, the number of repetitions is 100. Afterwards, we calculate the overall stability for each number of neighbours using ECC (the average ECS calculated between every pair of resulting modules). The value with the optimal distribution (high ECC median and low ECC IQR) is then selected.

On the chosen configuration, the partitions are then grouped by the number of clusters. We calculate the ECC of all partitions having a specific number of clusters (*k*). Based on the average score and the frequency of *k*, we select the stable modules. By default, we define *k* as a stable number of gene clusters if the average ECC is greater than 0.9 (which indicates high stability, given that the maximum is 1) and appears in at least 30 partitions (to ensure we have a sample size that would make the assessment relevant).

We opted to use Element-Centric Stability as the default score for comparing two clustering outputs based on benchmarks demonstrating better robustness in a multitude of scenarios that include randomised changes of membership, significant shifts in cluster sizes, and varying number of clusters (Gates et al. 2019).

### Module Aggregate Expression and Associated Population

Given a module, we define its expression as the cell-wise average of the expressions from each gene. Consequently, high aggregate values correspond to cells where genes are either consistently expressed across the entire set or highly expressed in a notable subpopulation. We proceed to scale the aggregate values between zero and one using min-max scaling.

The resulting aggregate vectors can be used to define the population of cells that are described by each cluster of genes. By default, we define the population using the cells that have a scaled aggregate expression above 0.5.

We note that the voting scheme functionality from Starlng shows great flexibility in setting the conditions to define the module-associated population. Before aggregation, cells can be pre-filtered using per-gene expression thresholds (either fixed or adaptively using top percentiles). Moreover, a lower bound for the number of co-expressed genes can be set.

### Module Filtering

Grouping several genes that show individual expression patterns does not guarantee that the entire module retains the same characteristics. Thus, we introduce a post-processing step of the modules in the Starlng pipeline that flags outlier and redundant modules.

We first identify the populations associated with each module and generate a summary based on the pseudotime interquartile range (IQR) and the UMAP median intra-population distance.

Based on this summary, we calculate the modified Z-score (using MAD) and flag as outliers the modules whose score exceeds 3.5. We recall that the formula of the modified Z-score is $\frac{0.6745\cdot\left( x_{i}-\tilde{x} \right)}{MAD}$, where $MAD=\text{median}\left( x_{i}-\tilde{x} \right)$ and $\tilde{x}$ is the median of the distribution.

Moreover, we provide an optional threshold that relies on the assumption that the dataset contains more than one homogenous cell population (this assumption is important; if the dataset contains a single celltype, then the threshold-based outlier detection becomes invalid and should be bypassed). We define a baseline by calculating the pseudotime IQR and the UMAP median distance using all the cells from the data. If a module lies within 85% of the values of the baseline, we label it as an outlier, given its behaviour is not significantly different from a random assignment of population.

We also introduce an intermediary category for redundant modules. The phenomenon we observed was that some modules had a very specific population, whereas other non-outlier modules spanned larger areas formed by multiple specific groups.

To encourage the analysis of the first type of modules, we introduced a greedy heuristic where we order the modules based on their variability (defined by either the pseudotime IQR or the UMAP median distance). The first quarter (based on this ordering) is automatically considered non-redundant and will be used to build the baseline for the following steps.

We iterate through this sorting, and, for each module (we will name it X for convenience), we define the specific population that would be introduced by it in comparison with the area that was already covered by the previous modules. If at least 50% of the cells expressing X belong to this specific population (in other words, if at least half of the module association is uniquely distinct from the previous modules), we label the module as non-redundant.

In the second phase, we define a metric that calculates the harmonic mean between the percentage of unique cells and the percentage of added cells with respect to the entire dataset. The aim of this metric is to reward modules that introduce numerous unique cells while penalising the modules that have a high overlap percentage. We calculate this metric for every module. We define the threshold as the median of the scores associated with the non-redundant modules. A module will be relabelled as non-redundant if its score exceeds this threshold.

The final phase targets redundant modules that have an almost complete overlap with another non-redundant module. They virtually describe the same population, but one was discarded because of the inclusion of the other. If an overlap percentage exceeds 95%, the module will be relabeled as non-redundant.

The algorithm will return the outlier-redundant labelling for each module, along with summaries of the evolution of the coverage across the increasing variance.

We analysed the effects of the outlier-detection IQR percentage and the module-overlap threshold in Suppl. Section 10.

### Identification of Hub Genes

In Starlng, we employed a scoring method that determines the importance of each gene inside its module. The score is defined as the product of two components. For the first component, the similarity graph created in the second step of the gene clustering pipeline is used to define the links between genes. For each feature, we sum up the weights of its incident edges (given the SNN graph is undirected, the edge directionality is not considered), and we normalise the values using the maximum score from the module.

The second component compares the module-induced population to the gene-induced population of cells. The default scaled threshold for the module population is 0.5, as stated above. For each gene, we used the top 50% expressed cells (ranked by their expression) to define its population. We calculate the F1 score to maximise the overlap and minimise the specific differences simultaneously.

The final scores range between zero and one. Higher values indicate that the gene is well connected inside the module graph and reproduces the module population with high fidelity.

### Identification of Transcription Factors

For the functional interpretation of the discovered modules, we have used the *gprofiler2* (Kolberg et al. 2020) enrichment analysis tool. Our focus was on matching transcription factors for each gene cluster using the TRANSFAC database (Matys et al. 2006).

The associations were grouped by the name of the transcription factors (so different motifs were aggregated). The TFs with similar names (for example, *Hnf4a* and *Hnf4-alpha*) were concatenated as well.

Next, we used the trajectory graph to determine the transition between gene modules and, subsequently, the potential interaction between transcription factors. For each module, we identify the centroid of its associated cell population and determine the nearest vertex on the trajectory graph. The unmapped vertices are then removed; while removing them, we draw additional edges between their nearest nodes. This allows for the creation of cycles inside the graph. To address this, we analyse each triangle and remove the edge between the nodes that are farthest with respect to the geodesic distance. Finally, we add edges between modules that are associated with the same vertex.

This module transition graph is translated into a TF transition graph using the module-TF mapping created after the enrichment analysis.

### Datasets with predefined regulatory interactions

To compare the output of the gene clustering algorithms against a simulated ground truth, we created multiple synthetic datasets using the SERGIO framework (Dibaeinia, Sinha 2020). Given a GRN, SERGIO simulates the dynamics between transcription factors using stochastic differential equations. The dynamic function can be instantiated into multiple cell types depending on the number of desired clusters provided by the user. The resulting count matrix can be perturbed using three types of technical noise: introducing outlier genes by shifting their expression by a given factor; adding variability that mimics sequencing depth differences; and removing random counts from the matrix. Moreover, the inferred dynamics can be used in the framework to simulate differentiation across different celltypes, given a trajectory.

Our GRN input was extracted from the SCENIC run on the MASLD/MASH Immune dataset. Each gene module will be defined as the list of genes that are regulated by a transcription factor. To avoid overlap between clusters, each gene was associated with the TF with the highest regulatory weight.

We provided four GRN inputs to SERGIO by varying the number of TFs (and subsequently the number of modules): five, 10, 15 and 20. The Transcription Factors were selected randomly.

For each GRN, we generated four datasets, combining clean / noisy count matrices with no differentiation / transition between cell states. To avoid generating an artificial dataset where each cluster associates with exactly one node, we set the number of cell types to 60% of the number of target gene clusters. Each celltype consisted of 400 cells. All parameters were kept at the defaults suggested by the SERGIO tutorial.

Synthetic datasets can illustrate the potential behaviour of the pipeline, but they cannot fully reproduce the complexity of biological data. Because the GRN used in the SERGIO simulations was inferred from the MASLD/MASH dataset, these simulations assess recovery relative to that inferred network rather than to independently established biological ground truth.

### Gene Clustering Methods from Known Literature

In this paper, Starlng was compared against four other methods that are used to infer gene modules. We will briefly present each method, along with the used parameters and describe the approach to ranking the genes inside a module.

Hotspot (DeTomaso, Yosef 2021) is a framework that creates modules based on similarity and autocorrelation. The expression matrix is converted to a similarity graph using kNN. Compared to Monocle3 and Starlng, Hotspot uses this graph, instead of the trajectory graph, to determine local autocorrelation using Geary’s C measure (Geary 1954). The genes are filtered based on this score; subsequently, hierarchical clustering is applied on the pairwise correlation matrix.

We used the parameters shown in the framework tutorial: genes with adjusted local autocorrelation p-value (using FDR) above 0.05 are discarded. Modules should have at least 20 genes. We calculated the weight of each gene using the C autocorrelation score.

SCENIC (Aibar et al. 2017) is a popular framework that has been used to infer major regulatory interactions. The method first applies GENIE3 on the expression matrix to determine the interaction strength between genes and transcription factors. Using an importance measure threshold, the authors filter the uninformative links and thus create the modules. Only clusters with at least 20 genes are retained. A final filtering is applied by keeping the modules with transcription factors whose motif is enriched.

We used the command line version of the pyScenic tool with default parameters. The weights of the genes were calculated based on the regulatory strength.

hdWGCNA (Morabito et al. 2023) uses expression correlation to cluster the genes. The first step is to calculate the Spearman correlation between each pair of expression vectors. The values are then exponentiated and used to calculate the topological overlap matrix (TOM) which describes the neighbourhood matching of the genes. The TOM network is then clustered using the Dynamic Tree Cut algorithm (Langfelder, Zhang, Horvarth 2007), forming the gene modules. The weight of a gene inside a module is calculated as the correlation between its expression and the module eigengene.

We used the recommended parameters from the introductory vignette of the package. Genes that have less than five percent of cells with positive expression are discarded.

PAGA (Wolf et al. 2019) and CellRank (Lange et al. 2022) are two methods developed by the Theis Lab that use an inferred trajectory to determine gene modules. PAGA relies on creating the kNN similarity graph between cells (approach used in Monocle3 and Hotspot as well). The graph is then clustered using Louvain (Blondel et al. 2008), a community detection method. The framework creates a supergraph where each node is associated with a cluster, and the edges are weighted by the intercluster connectivity. The pseudotime is thus defined as the random-walk distance between a vertex and the selected root node. CellRank is then used to identify genes that differentiate across the determined lineage. Their expression is smoothed using GAM, z-transformed, and then the genes are clustered using the PhenoGraph pipeline, as described above.

We used 30 nearest neighbours to build the graph on the 30-dimensional Principal Component Embedding. We assisted in the selection of the root node using the Starlng automatic recommendation approach. Top 2000 Highly Variable genes were provided as input for clustering. The genes were clustered using the Leiden algorithm with a resolution value of 1. The weight of the gene is set to be proportional to its average expression.

We note that among the benchmarked literature methods, only PAGA + CellRank utilises a pseudotime trajectory for clustering genes.

### Comparison Methodology

We proposed five objective metrics to evaluate the behaviour of the five gene clustering algorithms on real datasets, and two metrics for synthetic datasets with known regulatory interactions.

The first objective covers the reliability of gene clustering based on the proportion of modules that are labelled as non-redundant after filtering. Low percentage of selected modules indicates presence of noise in the partition and, subsequently, in the downstream analysis.

The second score measures the data explainability rate. We must ensure that the dataset (and each cell type) is well represented by the identified gene modules. Hence, we calculate the total coverage as the percentage of cells that belong in at least one module-defined population.

The third score describes the compactness of the inferred cell populations. Broader areas impede focused downstream analysis; thus, we penalise methods based on the maximum variance (described by pseudotime IQR) of a module-associated cell population.

Next, we evaluate the quality of transition between neighbouring modules across the pseudotime trajectory. For the transition between modules, we calculated the jumps that each cluster should make towards its neighbour on the pseudotime landscape. Positive jumps indicate the presence of gaps, while negative jumps suggest that the pseudotime ranges of the two gene groups are partially overlapping. Ideally, there should be no gaps between populations; in case the modules overlay, the expected behaviour is that the two ranges are very similar. For two non-overlapping modules X and Y (the pseudotime median of X is lower than Y’s), the jump is calculated as the difference between the pseudotime Q1 of Y and the pseudotime Q3 of X. In the overlapping case, we calculate the average specific distances between corresponding quartiles.

Finally, we evaluate each method based on the number of statistically enriched biological processes within the resulting modules. The output of the enrichment analysis can be highly influenced by the size of the input set of genes. Therefore, to make the results comparable, we selected the top 10 hub genes from each module. Moreover, in the comparison context, an undesired effect is that the number of modules directly impacts the number of terms. Hence, the methods that output more clusters will be favoured. To address this, we selected the top five modules ordered for each method by the number of enriched terms. The enrichment analysis was performed against the GO:BP and TRANSFAC databases. We removed generic terms with a term size greater than 500.

When a dataset with predefined regulatory interactions is provided, we compare the methods using two metrics. The first relates to similarity. Our choice is to use the ECS score (Gates et al. 2019) to calculate the similarity between the simulated ground truth and the clustering output. The score ranges between zero (completely disjoint clustering, where every cell pair resides in different clusters) and one (perfect match).

The second metric relates to the percentage of retained genes. Each clustering method suggests a preprocessing step that discards uninformative genes: Starlng and Hotspot use autocorrelation patterns; CellRank uses by default only highly variable genes. Moreover, the procedure of some methods imposes that ambiguous genes are clustered in an outlier module, which should be discarded. Because all genes in our synthetic dataset are explicitly designed to be informative, methods are penalised proportionally to the percentage of genes they discard.

All seven metrics can be used to evaluate the performance of a method, but they can act as a proxy for drawing up a comparison analysis between several methods. In this scenario, we rank the frameworks for each score and create an overall assessment based on the sum of ranks. Lower values of the sum indicate that the respective method outperforms the others with respect to the proposed criteria.

### Runtime Benchmarking

To benchmark the runtime of the Starlng pipeline, we have used the expression profile of the MASLD/MASH immune cells. We used Zinbwave to fit the distribution of expression to a zero-inflated negative binomial (ZINB) model. This model is then used to extrapolate and simulate counts for a larger number of cells. In our context, we generated synthetic datasets with a number of cells ranging from 10 000 to 100 000.

We have used the top 3000 Highly Variable genes, identified using the *SCTransform* function from the Seurat package.

When building the ZINB model, we set the number of latent populations (K) to three.

## Section 2. Starlng Shiny App

The aim of the Starlng app is to provide tools to analyse and explore data irrespective of the computing background. The user can visualise the distribution of both metadata and gene expression patterns in a low-dimensional space. For multiple genes, we employ a voting scheme that highlights the cells that show coexpression, with the option of controlling the flexibility of filtering.

Secondly, the app contains powerful tools that can be operated in real-time. For clustering, the user can change the autocorrelation filter and the parameters involved in the stability assessment. On the chosen partitioning of the genes, the visualisation of the modules is accompanied by an automatic filtering of the clusters. Starlng identifies outlier modules and redundant modules based on the IQR of the pseudotime: for the first, the values are outside a dataset-specific range; for the second, the described cell population has already been included by other gene modules with lower values.

Moreover, the modules are automatically ordered based on their position on the trajectory using the pseudotime median. Their biological functions can be assessed using the enrichment tools available in the app.

## Section 3. Usable, redundant and outlier modules

One of the features of the Starlng framework is the automatic filtering of the genes based on outlier or redundant behaviour. To test its effectiveness, we conducted a part of the analysis steps on the entire set of modules. Suppl. Fig. 1C shows that the UMAP distribution of the gene is heavily impacted by the presence of the outlier.

This effect is present on the pseudotime level as well. Suppl. Fig. 1E helps us identify outliers such as 36, 30, 35. Their presence decreases the degree of separation between the three main groups that were originally identified in the default run.

Removing the filtering decreases the quality of the evaluation of how the gene modules describe the transitioning across the pseudotime trajectory. This can be seen by looking at the lack of pattern in expression in the aforementioned modules in Suppl. Fig. 1D.

While these arguments mostly refer to the outlier modules, we remove by default the redundant gene clusters as well to promote an analysis that is built on focused, population-specific gene groups.

## Section 4. Generalisability and Validation Across Multiple Datasets

To demonstrate the generalisability and robustness of Starlng across multiple datasets from different conditions, we extended our analysis to two subsets of the Cao et al. Human cell atlas of fetal gene expression (Cao et al. 2020). We selected the Hepatoblast cells from the Liver and the Acinar, Ductal, and Islet endocrine from Pancreas.

For the Pancreas dataset, Starlng identified a 15-Shared Nearest Neighbour graph as the optimal configuration in terms of stability (Suppl. Fig. 9A). The highest number of stable modules, 18, was selected for analysis (Suppl. Fig. 9B). Applying module filtering revealed a number of 10 selected gene clusters. The cell populations associated with each module are illustrated in Suppl. Fig. 10.

Projecting these modules on a 2D UMAP space shows a clear separation between cluster 2 and the other genes (Suppl. Fig. 9C). The top 5 hub genes of this module (*MKI67, CENPE, CENPF, TPX2, TOP2A*) reveal that Starlng successfully separated cell-cycle genes (Tirosh et al. 2016) from other modules.

The violin plot from Suppl. Fig. 9D shows an incremental transition between modules across the pseudotime trajectory, indicating a good coverage of the data. This is confirmed by Suppl. Fig. 9E, where each cell cluster is well-represented by at least one module. Conversely, we note that each module (except module six) describes a very specific population, mapped to a single cluster.

To functionally interpret the filtered modules, we performed enrichment analysis against GO:BP (top 25 terms shown in Suppl. Fig. 9F) and TRANSFAC databases (top 10 terms shown in Suppl. Fig. 3A, third panel).

Starlng successfully identified the major pancreatic cell types and grouped them into distinct functional modules based on characteristic hub genes. The algorithm accurately mapped beta cells (Module 17) through *NEUROD2, SUSD2, CADM2, MDK*, and *EYA2*; delta cells (Module 11) through *SST, CRH*, and *NLGN1*; epsilon cells (Module 15) through *GHRL, FGF12*, and *FGF14*; and acinar cells (Module six) through *CPA1, CPA2, CTRB1, CTRB2*, and *CELA3A* (Craig-Schapiro et al. 2025).

The enrichment results further supported these cell-type assignments. For example, the delta cell module was enriched for terms related to synapse assembly, synapse organization, and cell junction organization (Arrojo e Drigo et al. 2019), while the beta cell module was enriched for neurodevelopmental processes like behavioural fear and defence response (Rorsman, Ashcroft 2018). In addition, the acinar cell module was associated with cellular responses to metal ions and detoxification processes in conjunction with the enrichment of *MTF-1* (Andrews 2001). Overall, these results demonstrate that Starlng can recover biologically meaningful cell-type-specific modules, pathways, and regulatory signals from pancreatic tissue.

To evaluate the five clustering methods on the Pancreas dataset, we use Suppl. Fig. 11 as a starting point. Panel A visually highlights stark differences in cluster numbers and pseudotime coverage, while panel B reveals significant differences in signal-to-noise ratios across the workflows: Starlng and Hotspot show the clearest transition across the pseudotime trajectory.

To quantify the visual assessment, we calculate the values of the proposed five comparison metrics. The left panel from Suppl. Fig. 5A shows that Starlng retains 56% of the initial set of gene clusters. While hdWGCNA retains 60%, it identified only five modules compared to Starlng’s 18. Starlng achieved 83% cell coverage using the filtered modules (Suppl. Fig. 5B, left panel). Hotspot provides higher coverage (94%); however, Starlng holds the lowest pseudotime variance for the module-induced populations (maximum IQR of 5.96).

Starlng ranked first in trajectory continuity (Suppl. Fig. 12B). In functional enrichment analysis (GO:BP) using the top 10 hub genes per module, Starlng achieved the third-highest number of enriched terms behind Hotspot and PAGA + CellRank (Suppl. Fig. 5C).

The cumulative evaluation ranking is presented in Suppl. Fig. 9G, which demonstrates that Starlng achieved the best overall performance.

For the Liver dataset, Starlng identified a 10-Shared Nearest Neighbour graph as the optimal configuration in terms of stability (Suppl. Fig. 13A). The highest number of stable modules, 12, was selected for analysis (Suppl. Fig. 13B). Applying module filtering revealed a number of seven selected gene clusters. The cell populations associated with each module are illustrated in Suppl. Fig. 14.

Projecting these modules on a 2D UMAP space shows a clear separation between clusters one and nine and the other genes (Suppl. Fig. 13C). The violin plot from Suppl. Fig. 13D shows an incremental transition between modules across the pseudotime trajectory, indicating a good coverage of the data. The plot suggests a clustering of modules 9 and 1, and of modules three, four and seven, respectively. This is confirmed by Suppl. Fig. 13E, where each cell cluster is well-represented by at least one module.

To functionally interpret the filtered modules, we performed enrichment analysis against GO:BP (top 25 terms shown in Suppl. Fig. 13F) and TRANSFAC databases (top 10 terms shown in Suppl. Fig. 3A, second panel).

The hepatocyte identity was captured by Starlng modules. Module one contains established hepatocyte genes such as *ZBTB20* (To et al. 2021), *RORA* (Han et al. 2019), and *DPYD* (Cavalli et al. 2020). The functional analysis strengthens the claim; we note the presence of uracil, thymine metabolism, pyrimidine catabolism, as well as the enrichment of liver-associated transcription factors *C/EBP* and *C/EBP-beta* (Lee et al. 2012).

Given that most of the analysed fetal liver dataset is linked to Hepatoblasts, the Starlng module filtering can be partially overridden (we emphasise that this is a corner case rather than a general rule). We notice that module two contains hub genes that were confirmed in the fetal atlas manuscript as being strongly linked to the hepatoblast signature: *AFP, ALB, APOC3, APOA1, APOA2, AMBP* (Cao et al. 2020).

In addition, Modules three and four recovered a cholangiocyte-associated transcriptional program consistent with the epithelial plasticity and hepatocyte-to-cholangiocyte transitions described in MASLD/MASH (Gribben et al. 2024). These modules contained several reported cholangiocyte-like hepatocyte markers, including *DCDC2, CDH6, and BICC1* (Module three), as well as *SOX4* and *SPP1* (Module four), suggesting that Starlng captures transcriptional states associated with biliary differentiation and cellular plasticity.

To evaluate the five clustering methods on the Liver dataset, we use Suppl. Fig. 15 as a starting point. The observations from the Pancreas dataset are recapitulated in this context as well.

To quantify the visual assessment, we calculate the values of the proposed five comparison metrics. The right panel from Suppl. Fig. 5A shows that Starlng retains the highest percentage (58%) of the initial set of gene clusters. Starlng achieved 38% cell coverage using the filtered modules (Suppl. Fig. 5B, right panel). PAGA + CellRank provides the highest coverage (64%); however, Starlng holds the second-lowest pseudotime variance for the module-induced populations (maximum IQR of 3.47, compared to the hdWGCNA maximum of 1.89).

Starlng ranked second in trajectory continuity (Suppl. Fig. 16B), which was heavily impacted by the gaps between modules eight to 11 and 11 to nine (Suppl. Fig. 16A). In functional enrichment analysis (GO:BP) using the top 10 hub genes per module, Starlng achieved the third-highest number of enriched terms behind PAGA + CellRank and Hotspot (Suppl. Fig. 5C).

The cumulative evaluation ranking is presented in Suppl. Fig. 13G, which shows that Starlng achieved the second-best overall performance. We note that the cumulative score is within one rank of the best-performing method, Hotspot.

## Section 5. The impact of the Moran’s I filtering threshold

Even if the range of the Moran’s I test values is known, balancing the signal-to-noise ratio and the number of genes poses the challenge of selecting an appropriate threshold. The aim of including Starlng with low filtering is to display the potential negative impact of including genes with an autocorrelation pattern close to the null model.

Based on the stability assessment presented in Suppl. Fig. 17A, B, we have chosen 40 nearest neighbours as the stable parameter configuration and 41 as the number of gene modules. We repeated the same procedure as in the default experiment, first creating the low-dimensional representation of the genes from the filtered modules, as shown in Suppl. Fig. 17C. We remark how the module separation is less clear compared to the stricter filtering. This is noticeable in Suppl. Fig. 17D as well: the violin distribution of the pseudotime indicates that the module filtering does not entirely discard clusters with high variance (as 35 or 39). Moreover, the grouping effect seen on the default run is more difficult to capture, except for the 33-26 group of modules that describe the late population.

Suppl. Fig. 17E presents that the modules cover a significant proportion of the existing cells. Firstly, a noticeable number of modules do not display any expression pattern and cannot be associated with any of the cell clusters. Secondly, some areas are no longer well-represented by the modules. Suppl. Fig. 17E shows that cluster nine is associated only to the gene modules with a very low specificity, 35 and 39. Furthermore, Suppl. Fig. 4B (fifth panel) shows that the signal in the upper half population on the pseudotime ordering has been diluted.

Based on these results and in comparison with the default run, we conclude that lowering Moran’s I threshold could be associated with introduction of noise and loss of interpretation and coverage of the cell populations.

## Section 6. Comparison with cell-cluster-defined gene sets

Cell-level clustering usually assigns cells to discrete groups, whereas gene-module approaches identify sets of genes whose activity can span overlapping cell populations. Starlng focuses on stability-aware, trajectory-guided gene-module discovery. To illustrate how this differs from conventional gene selection, we compared Starlng modules with two representative gene sets: differentially expressed genes and highly variable genes (HVGs). This comparison is based on the neutrophil subset of the MASLD/MASH dataset. The marker genes were obtained by applying the Wilcoxon Rank Sum test against the rest of the population. Figure 1D shows that modules 15 and 25 contain the autocorrelated genes that most accurately represent this lineage. The HVGs are determined using the VST method from the Seurat framework.

Suppl. Fig. 18A shows the mapping between the two gene sets, attempting to associate log-fold change (LFC) with autocorrelation. For high values, we note that all marker genes belong to one of the two modules (identified using orange - 15 and blue - 25). Lowering the log-fold values introduces marker genes that either are clustered in a different module (green colour) or have a low autocorrelation score and have not been included in the analysis (pink colour). These two groups form a majority for the lower range of log-fold change values, where usually the threshold is set. Moreover, the line from the same plot describes the evolution of the probability of a marker gene being assigned to the target modules in relation to the descending trend of the LFC. The results show that this probability is already worse than the random chance for a log-fold change value of two.

The overall trend is further captured in Suppl. Fig. 18B, which illustrates the LFC for each of the four groups described above. Despite modules 15 and 25 having overall greater values, we note the presence of a large group of genes with similar LFC (between 2.5 and 5.0), but no clear autocorrelation pattern. Moreover, some genes clustered in the two target modules (especially 15) are positioned in the lower spectrum of the LFC (between zero and one).

This remark is more evident in the comparison with the HVGs. The right panel from Suppl. Fig. 18B shows that there is no clear separation between autocorrelated and trajectory-dispersed genes in terms of the variability of expression.

These remarks aim to underline the discrepancies between the trajectory-guided modules and the gene sets derived at cell-cluster level. They also suggest that considering gene properties beyond differential expression or variance can provide complementary information when selecting genes for downstream functional interpretation.

## Section 7. Assessing Starlng Robustness

We evaluate the robustness of the outputs of the Starlng framework using three types of perturbation. In the first experiment, we subsample the cells with a percentage varying between 0.95 and 0.1. In the second experiment, we added noise to the normalised expression matrix. The noise follows the Gaussian distribution centred at zero and with the standard deviation varying between 0.1 and two. Both experiments were run ten times with different random seeds. The third assessment is performed by analysing the sensitivity to changes in the initial random state.

We will compare the results using the Element-Centric Similarity and Consistency metrics, as they have been proven to be less affected by biases induced by partition characteristics.

These experiments were conducted on the MASLD/MASH immune dataset. The comparison will be done with respect to the partitions with a stable number of modules.

Suppl. Fig. 19A shows the overall clustering consistency for various subsampling fractions. The results indicate that the clusters have a similarity with a median of 0.816 for a quarter of the initial number of cells. The similarity drops when only 10% are used, but only to a median of 0.680, which still indicates relevant overlap.

The results of the second experiment are illustrated in Suppl. Fig. 19B. We note that for a standard deviation of 0.25 (which is close to the median standard deviation of the expression distributions of the genes from the data), the clustering remains similar to the original output, with a median ECS of 0.81. The results drop noticeably to a median ECS of 0.5 when the standard deviation of the noise is increased to one. This is, however, expected, given that the standard deviation of the added noise is four times higher than that present in the original data.

Finally, panel C from Suppl. Fig. 19 show the overall consistency between multiple runs of the first two experiments with different random seeds. We observe a very similar descending pattern for both cases. This illustrates that Starlng generates stable outputs even when an aggressive subsampling (left figure) is applied, irrespective of the state of the random seed. On the other hand, introducing too much noise in the data (right figure) not only erases the initial assignment of the cells, but drastically impacts the separability of cell groups.

## Section 8. Computational Efficiency and Runtime Benchmarks

We evaluated the scalability of the Starlng framework by running the default configuration (except for the autocorrelation threshold, which was lowered from 0.1 to zero) on a synthetic dataset with size ranging from 10 000 to 100 000 and 3000 genes. The experiment was run on 30 parallel threads.

Suppl. Fig. 1F showcases the linear relationship between runtime and the size of the dataset. This is expected, as the majority of the steps have a time complexity proportional to the number of genes. The identification of feature loading represents the main exception to this behaviour, as the initial PCA is applied to the entire matrix. For the largest dataset, 45 minutes were required to execute the whole pipeline. Suppl. Fig. 1G highlights the optimisation achieved by parallelising the framework. Increasing the number of cores from one to five already achieves a two-fold improvement in runtime.

We have attempted to measure the amount of memory used during execution. Suppl. Fig. 1H shows that there is no direct relationship between the number of cells and the memory used. The presence of spikes is correlated to the autocorrelation test functionality from the Monocle3 package and advises using fewer cores on personal machines when running Starlng on a moderately large dataset. We note that this memory evaluation is not entirely precise and relies on querying the system-wide usage every five seconds.

## Section 9. Software Dependencies and Versioning

The trajectory graph, pseudotime ordering, and identification of auto-correlated genes were performed using the Monocle3 package v1.3.7 (Cao et al. 2019).

The feature loadings of the filtered genes were determined using prcomp (base R package v4.4.0). The nearest neighbour graph was calculated using the RANN package v2.6.1 (Jefferis et al. 2013), converted to an igraph object v2.0.3 (Csárdi et al. 2024) and clustered using the R implementation of the Leiden (Traag, Waltman, Van Eck 2019) algorithm (leidenbase package v0.1.30 (Ewing 2022)). UMAP (McInnes et al. 2018) was used for the 2D representation of genes via the uwot package v0.2.2.

The objects used in the Shiny application (Chang et al. 2024) were stored using the qs2 package v0.26.3 (Ching 2024). The expression matrix was saved using the rhdf5 R package v2.48.0 (Fischer, Smith, Pau 2017). For visualisation, we used ggplot2 v3.5.1 (Wickham 2016) and ComplexHeatmap v2.20.0 (Gu, Eils, Schlesner 2016) packages. The tables were rendered using the DT package v0.33 (Xie et al. 2015). The enrichment analysis was performed using the gprofiler2 package v0.2.3 (Kolberg et al. 2020).

. For the gene clustering methods that were used for comparison, we employed the following versions: SCENIC – pyScenic, GitHub commit 06bafba412792f6efa5a552a23bb221cc3bdea1b; hdWGCNA – R package v0.4.11; Hotspot – Python package v1.1.3; CellRank – Python package v1.1.3.

For benchmarking, we have applied the zinbwave R package v1.26.0 (Risso et al. 2018) to generate synthetic data. To compare the gene clustering outputs to a synthetic ground truth we generated 16 datasets using the SERGIO Python (Dibaeinia, Sinha 2020) using the latest version from the GitHub repository (commit a6190b74425112834c8fa9b4b6157d9cb3d1ab88).

## Section 10. Sensitivity Analysis of Parameters

The quality of results generated by Starlng depends on the choice of parameters used throughout the pipeline. Although dataset characteristics may influence the optimal settings, it is also important to identify parameter ranges that can reduce result quality. We investigated the sensitivity of several parameters at different stages of the framework in an ablation study using the MASLD/MASH dataset. The findings should therefore be interpreted in the context of this dataset.

First, we evaluated the number of nodes, scaled by log10(number of cells), used to construct the trajectory graph. We varied this number from five to 100 and calculated pseudotime using Starlng’s recommended starting point. The trajectory changed substantially at lower node counts but began to converge as the number of nodes increased. Suppl. Fig. 20A illustrates this pattern: pseudotime estimates from 5 and 10 nodes show areas of considerable discrepancy, whereas estimates from 90 and 100 nodes have a clear linear relationship. To quantify agreement between pseudotime vectors, we used four metrics: Spearman’s rank correlation, Earth Mover’s Distance (EMD; Rubner, Tomasi, Guibas, 1998), the R-squared of a linear regression, and the Jaccard similarity index (JSI) between sets of spatially autocorrelated genes (Moran’s I > 0.1). The metrics in Suppl. Fig. 20B indicate greater pseudotime stability at higher node counts. Although a large number of nodes could potentially overfit the trajectory structure, this is generally mitigated by the pruning mechanism in Monocle3.

We also evaluated how many iterations of the minimum spanning tree (MST) procedure were required to obtain a stable trajectory. Suppl. Fig. 20C suggests that the results converge rapidly.

Next, we tested whether selecting a node at either end of the graph diameter maximises the pseudotime range. We evaluated each node on the main trajectory as a candidate starting point for pseudotime ordering. In Suppl. Fig. 20D, point size represents the maximum pseudotime (equivalent to the range) and colour represents the mean PCA distance within pseudotime bins. Both measures support the same conclusion: moving away from the trajectory leaves reduces the pseudotime range -by nearly half as we approach the middle island- and increases the local sparsity of cells with similar pseudotime values.

Finally, we evaluated two parameters used in gene-module filtering. The first controls outlier-detection sensitivity by scaling the pseudotime-IQR and UMAP-distance thresholds by the same percentage. We varied this percentage from five percent to 100%. As shown in Suppl. Fig. 20E, lower percentages classified more modules as outliers. From 20% onwards, the number of outlier modules decreased substantially, with most of the change corresponding to an increase in redundant modules. The distribution stabilised at approximately 60%. Suppl. Fig. 20F shows the median within-module UMAP distance for each module category across these thresholds. The distances between outlier, redundant and filtered modules are most distinct and representative in the stable range above 60%.

We also assessed sensitivity to the overlap threshold, which determines whether a redundant module can be classified as filtered based on its overlap with an already filtered module. We varied this threshold from 0.05 to 1.00. Suppl. Fig. 20G shows the median within-module UMAP distance across thresholds. The distances stabilise when the threshold is approximately 0.8 or higher; in this range, filtered modules have a lower median within-module UMAP distance, consistent with greater spatial compactness in the associated cell populations.

## Section 11. Integration with other frameworks

We aim to leverage the technology-agnostic approach of the gene-clustering pipeline and thus to improve the accessibility of the Starlng package to most of the users. Given that the Pseudotime inference is calculated using the Monocle3 package (Cao et al. 2019), our challenge was to create utility functions that smooth the transition between other packages and Starlng.

By default, Starlng only requires a normalised gene-by-cell expression matrix to perform the stability assessment and generate the interactive Shiny App. This makes a strong argument for portability and ease of integration with other frameworks such as Seurat (Hao et al. 2024). We promote cross-compatibility with other programming languages by enabling communication via HDF5-formatted files to store the matrices.

Moreover, Starlng provides a seamless integration with the ClustAssess (Shahsavari, Munteanu, Mohorianu 2022) package. Starlng accepts the ClustAssess Shiny App as a valid input to run the module analysis pipeline.

# Abbreviations

ECS – Element-Centric Similarity

ECC – Element-Centric Consistency

GO:BP – Gene Ontology: Biological Process

FDR – False Discovery Rate

GAM – Generalized Additive Model

GRN – Gene Regulatory Network

IQR – Inter-Quartile Range

JSI – Jaccard Similarity Index

kNN – k-Nearest Neighbours

MAD – Median Absolute Deviation

MASLD – Metabolic Dysfunction-Associated Steatotic Liver Disease

MASH – Metabolic Dysfunction-Associated Steatohepatitis

MST – Minimum Spanning Tree

PAGA – Partitioned Approximate Graph Abstraction

PCA – Principal Component Analysis

Q1, Q3 – First and third quartile

SimplePPT – Simple PrinciPal Tree structure

SNN – Shared Nearest Neighbour

TF – Transcription Factor

TOM – Topological Overlap Matrix

UMAP – Uniform Manifold Approximation and Projection

# Supplementary Figures

**Suppl. Fig. 1 Extended results on Starlng run** A. Boxplot showing the Element-Centric Consistency (ECC) score (y-axis) achieved by various numbers of nearest neighbours (x-axis) used to build the gene graph. This plot is used to select the optimal configuration of parameters. B. Bubbleplot showing the stability of each number of gene modules (x-axis) that is obtained, given the range of resolution values. The size of the point suggests the average ECC score. Unstable values (ECC < 0.9) were marked with very small size. The colour gradient indicates the variance of the ECC distribution using ECC. The y-axis shows the number of occurrences of the number of modules. The horizontal dashed line represents the lower stability threshold of 30. The unstable numbers of gene clusters are marked in red. C. 2D representation of all the genes, coloured by the modules identified by Starlng. Module 30 is a clear outlier that affects the interpretation of module groups. D. Bubbleplot showing the association between the stable 24 cell clusters identified by ClustAssess and all gene modules. The size of the point is proportional to the number of cells from the clusters that are identified by a module. The colour gradient indicates the strength of the scaled average expression of the genes belonging to the module. Multiple modules that were filtered out show no relevant expression patterns or association with a cell cluster (such as 35 or 30). E. Violin plot showing the distribution of pseudotime (y-axis) of all Starlng gene modules (x-axis). The presence of outlier modules (such as 30, 16) is noticeable and impacts the transition between groups. F. Boxplot presenting the execution runtime, measured in minutes (y-axis) of the Starlng framework on datasets with sizes ranging from 10,000 to 100,000 (x-axis). We notice a tight distribution and virtually linear scaling. G. Boxplot presenting the execution runtime, measured in minutes (y-axis) of the Starlng frameworks on datasets with 100,000 cells while changing the number of parallel threads (x-axis). We notice that the major speedup is achieved when increasing the number of parallel processes from 1 to 5. H. Boxplot presenting the memory usage, measured in GB (y-axis) when running Starlng on datasets with sizes ranging between 10,000 and 100,000 (x-axis). The red line displays the trend described by the maximum values achieved for each dataset size.

**Suppl. Fig. 2 Description of the module-specific cell population (MASLD/MASH)** 2D representation of the cell groups that are defined by each filtered Starlng module in the Gribben et al. 2024 MASLD/MASH Immune dataset. The colour gradient indicates the scaled average expression calculated at the gene cluster level. The cells with scaled values lower than 0.5 were discarded. The modules are ordered based on the median pseudotime values to better capture the trajectory.

**Suppl. Fig. 3 Transcription Factor Analysis** A. Bubbleplot showing the relationship between the TRANSFAC enriched terms (x-axis) and the filtered modules (y-axis). The size of the point is proportional to the number of genes used to identify a particular TF. The colour gradient showcases the number of hub genes (out of 15) that were involved in the enrichment. For each module, we select the top TFs, ordered by the number of associated genes. Three panels are presented: Gribben et al. 2024 MASLD/MASH Immune dataset (top), Cao et al. 2020 Liver (middle) and Cao et al. 2020 Pancreas (bottom). B. Network representation showing the interaction between TFs (triangles) and genes (diamonds). The colour of each gene represents its module. The links between TFs and genes are drawn if the gene is involved in the enrichment of the factor. The links between TFs are drawn if they are defined in the same module or in neighbouring modules. To reduce the complexity of the graph, we keep only the edges between consecutive TFs (based on the gene set size ordering). Three panels are presented: Gribben et al. 2024 MASLD/MASH Immune dataset (left), Cao et al. 2020 Liver (middle) and Cao et al. 2020 Pancreas (right).

**Suppl. Fig. 4 Module expression across pseudotime (MASLD/MASH)** The figure shows the evolution of the aggregate expression of the filtered modules identified by each gene clustering framework across the pseudotime trajectory on the Gribben et al. 2024 MASLD/MASH Immune dataset. Two representations are employed: A - smoothed expression (using GAM); each line corresponds to a module. The peak of the function denotes the pseudotime area that the module-associated population covers; B - Heatmap showing the pattern of expression of the genes that belong to the modules. Each row represents a gene, and the colourbar on the left reflects the gene modules. Each column represents a cell, and the colourbar on the bottom reflects the stable 24 clusters. The cells are ordered by the pseudotime values. The colour gradient represents cell-specific scaled expressions.

**Suppl. Fig. 5 Extended performance comparison between Starlng and other methods** Panels show a comprehensive evaluation of four metrics (out of five) across three datasets: Cao et al. 2020 Pancreas, Gribben et al. 2024 MASLD/MASH Immune and Cao et al. 2020 Liver. A. Barplot showing the percentages of filtered (yellow), redundant (blue) and outlier (red) modules. The number of clusters is displayed on the right of each barplot. The labels indicate the number of genes from each of the three categories. B. Line plot showing the cumulative coverage of cells from the gene modules (y-axis). On the x-axis, we display the maximum pseudotime IQR of the modules that are included in the analysis. Ideally, a method should show high coverage value using low-variance gene clusters. C. UpSet plots showing the intersection of significant enriched term sets from GO:BP (columns 1,3) and TRANSFAC (columns 2, 4). Comparisons are evaluated using either all filtered genes in the analysis (columns 1, 2) or the top 10 hub genes, with terms selected from the top 5 clusters based on term set size (columns 3, 4).

**Suppl. Fig. 6 Pseudotime-based comparison between Starlng and other methods (MASLD/MASH)** A. Boxplots showing the pseudotime distribution of filtered modules across methods, ordered by median pseudotime. B. Barplot illustrating pseudotime jumps between consecutive modules (ordered as in panel A). Negative values indicate a quartile decrease in the pseudotime distribution, with the total jump magnitude indicated above each distribution. C. Histogram showing the distribution of cells included in one or multiple modules. Cell inclusion is defined here using the IQR of the pseudotime distribution (not the aggregate expression). Zero overlaps denote the presence of gaps between modules. One overlap denotes cells unique to a specific module.

**Suppl. Fig. 7 Description of the synthetic datasets with known interactions** 2D UMAP representation of the datasets generated using SERGIO. The colours indicate the celltype identities. Each column is associated with a different number of simulated ground truth gene clusters (5, 10, 15, 20). Rows illustrate the inclusion of experimental confounding factors: steady-state counts without a pre-defined trajectory (Rows 1, 2) versus simulated continuous trajectories (Rows 3, 4), evaluated both without technical noise (Rows 1, 3) and with technical noise added to the raw count matrix (Rows 2, 4).

**Suppl. Fig. 8 Ground truth evaluation** A. Boxplots showing the clustering similarity (calculated using ECS) between the simulated ground truth modules and the output of the gene clustering frameworks. The x-axis is grouped based on the four different types of data, defined by all combinations between defining a trajectory and adding noise. For each group there are 4 different numbers of target clusters. B. Barplot showing the percentage of genes that were discarded in the preprocessing of the method (no stripes) or declared ambiguous and clustered in an outlier module (stripes). The x-axis follows the same principles from Panel A.

**Suppl. Fig. 9 Starlng results on Pancreas dataset** A. Boxplot showing the Element-Centric Consistency (ECC) score (y-axis) achieved by various numbers of nearest neighbours (x-axis) used to build the gene graph. This plot is used to select the optimal configuration of parameters. B. Bubbleplot showing the stability of each number of gene modules (x-axis) that is obtained, given the range of resolution values. The size of the point suggests the average ECC score. Unstable values (ECC < 0.9) were marked with very small size. The colour gradient indicates the variance of the ECC distribution using ECC. The y-axis shows the number of occurrences of the number of modules. The horizontal dashed line represents the lower stability threshold of 30. The unstable numbers of gene clusters are marked in red. C. 2D representation of the genes, coloured by the modules identified and filtered by Starlng. The UMAP was built on the feature loading calculated using PCA at the cell level. For each module, the names of its top 5 hub genes are displayed. Top 25% of the edges (ordered by the SNN weights) are shown. D. Violin plot showing the distribution of pseudotime (y-axis) of the filtered Starlng gene modules (x-axis). E. Bubbleplot showing the association between the Seurat clusters and the filtered gene modules. The size of the point is proportional to the number of cells from the clusters that are identified by a module. The colour gradient indicates the strength of the scaled average expression of the genes belonging to the module. F. Bubbleplot showing the modules that were used to identify the top 25 enriched GO:BP terms. The size of the point is proportional to the percentage of top 15 hub genes that are used to identify the term. The colour gradient reflects the pvalue significance (bright colour – low pvalue). G. Table showing the results of the overall comparison between Starlng and the other four gene clustering frameworks. Each cell contains the ranking that a method achieved for a particular metric, the actual value being mentioned in the parentheses. The median and sum rank dictate the overall ranking of the method. Starlng demonstrates the best performance on this dataset.

**Suppl. Fig. 10 Description of the module-specific cell population (Pancreas)** 2D representation of the cell groups that are defined by each filtered Starlng module in the Cao et al. 2020 Pancreas dataset. The colour gradient indicates the scaled average expression calculated at the gene cluster level. The cells with scaled values lower than 0.5 were discarded. The modules are ordered based on the median pseudotime values to better capture the trajectory.

**Suppl. Fig. 11 Module expression across pseudotime (Pancreas**) The figure shows the evolution of the aggregate expression of the filtered modules identified by each gene clustering framework across the pseudotime trajectory on the Cao et al. 2020 Pancreas dataset. Two representations are employed: A – smoothed expression (using GAM); each line corresponds to a module. The peak of the function denotes the pseudotime area that the module-associated population covers. B - Heatmap showing the pattern of expression of the genes that belong to the modules. Each row represents a gene, and the colourbar on the left reflects the gene modules. Each column represents a cell, and the colourbar on the bottom reflects the Seurat clusters. The cells are ordered by the pseudotime values. The colour gradient represents cell-specific scaled expressions.

**Suppl. Fig. 12 Pseudotime-based comparison between Starlng and other methods (Pancreas)** A. Boxplots showing the pseudotime distribution of filtered modules across methods, ordered by median pseudotime. B. Barplot illustrating pseudotime jumps between consecutive modules (ordered as in panel A). Negative values indicate a quartile decrease in the pseudotime distribution, with the total jump magnitude indicated above each distribution. C. Histogram showing the distribution of cells included in one or multiple modules. Cell inclusion is defined here using the IQR of the pseudotime distribution (not the aggregate expression). Zero overlaps denote the presence of gaps between modules. One overlap denotes cells unique to a specific module.

**Suppl. Fig. 13 Starlng results on Liver dataset** A. Boxplot showing the Element-Centric Consistency (ECC) score (y-axis) achieved by various numbers of nearest neighbours (x-axis) used to build the gene graph. This plot is used to select the optimal configuration of parameters. B. Bubbleplot showing the stability of each number of gene modules (x-axis) that is obtained, given the range of resolution values. The size of the point suggests the average ECC score. Unstable values (ECC < 0.9) were marked with very small size. The colour gradient indicates the variance of the ECC distribution using ECC. The y-axis shows the number of occurrences of the number of modules. The horizontal dashed line represents the lower stability threshold of 30. The unstable numbers of gene clusters are marked in red. C. 2D representation of the genes, coloured by the modules identified and filtered by Starlng. The UMAP was built on the feature loading calculated using PCA at the cell level. For each module, the names of its top 7 hub genes are displayed. Top 25% of the edges (ordered by the SNN weights) are shown. D. Violin plot showing the distribution of pseudotime (y-axis) of the filtered Starlng gene modules (x-axis). E. Bubbleplot showing the association between the Seurat clusters and the filtered gene modules. The size of the point is proportional to the number of cells from the clusters that are identified by a module. The colour gradient indicates the strength of the scaled average expression of the genes belonging to the module. F. Bubbleplot showing the modules that were used to identify the top 25 enriched GO:BP terms. The size of the point is proportional to the percentage of top 15 hub genes that are used to identify the term. The colour gradient reflects the pvalue significance (bright colour – low pvalue). G. Table showing the results of the overall comparison between Starlng and the other four gene clustering frameworks. Each cell contains the ranking that a method achieved for a particular metric, the actual value being mentioned in the parentheses. The median and sum rank dictate the overall ranking of the method. Starlng demonstrates the second best performance on this dataset.

**Suppl. Fig. 14 Description of the module-specific cell population (Liver)** 2D representation of the cell groups that are defined by each filtered Starlng module in the Cao et al. 2020 Liver dataset. The colour gradient indicates the scaled average expression calculated at the gene cluster level. The cells with scaled values lower than 0.5 were discarded. The modules are ordered based on the median pseudotime values to better capture the trajectory.

**Suppl. Fig. 15 Module expression across pseudotime (Liver**) The figure shows the evolution of the aggregate expression of the filtered modules identified by each gene clustering framework across the pseudotime trajectory on the Cao et al. 2020 Liver dataset. Two representations are employed: A – smoothed expression (using GAM); each line corresponds to a module. The peak of the function denotes the pseudotime area that the module-associated population covers. B - Heatmap showing the pattern of expression of the genes that belong to the modules. Each row represents a gene, and the colourbar on the left reflects the gene modules. Each column represents a cell, and the colourbar on the bottom reflects the Seurat clusters. The cells are ordered by the pseudotime values. The colour gradient represents cell-specific scaled expressions.

**Suppl. Fig. 16 Pseudotime-based comparison between Starlng and other methods (Liver)** A. Boxplots showing the pseudotime distribution of filtered modules across methods, ordered by median pseudotime. B. Barplot illustrating pseudotime jumps between consecutive modules (ordered as in panel A). Negative values indicate a quartile decrease in the pseudotime distribution, with the total jump magnitude indicated above each distribution. C. Histogram showing the distribution of cells included in one or multiple modules. Cell inclusion is defined here using the IQR of the pseudotime distribution (not the aggregate expression). Zero overlaps denote the presence of gaps between modules. One overlap denotes cells unique to a specific module.

**Suppl. Fig. 17 Running Starlng with a low Moran's I filter**. A. Boxplot showing the Element-Centric Consistency (ECC) score (y-axis) achieved by various numbers of nearest neighbours (x-axis) used to build the gene graph. B. Bubbleplot showing the stability of each number of gene modules (x-axis) that is obtained, given the range of resolution values. The size of the point suggests the average ECC score. Unstable values (ECC < 0.9) were marked with very small size. The colour gradient indicates the variance of the ECC distribution using ECC. The y-axis shows the number of occurrences of the number of modules. The horizontal dashed line represents the lower stability threshold of 30. The unstable numbers of gene clusters are marked in red. C. 2D representation of the genes, coloured by the modules identified and filtered by Starlng. The UMAP was built on the feature loading calculated using PCA at the cell level. For each module, the names of its top 2 hub genes are displayed. Top 25% of the edges (ordered by the SNN weights) are shown. Grouping of the modules is not as clear as it was shown in stricter filtering. D. Violin plot showing the distribution of pseudotime (y-axis) of the filtered Starlng gene modules (x-axis). A single grouping of the modules can be identified at the end of the pseudotime trajectory. E. Bubbleplot showing the association between the stable 24 cell clusters identified by ClustAssess and the filtered gene modules. The size of the point is proportional to the number of cells from the clusters that are identified by a module. The colour gradient indicates the strength of the scaled average expression of the genes belonging to the module. Multiple modules show no clear association pattern with any of the groups. The trajectory across the dataset is not clearly described. F. Bubbleplot showing the modules that were used to identify the top 25 enriched GO:BP terms. The size of the point is proportional to the percentage of top 15 hub genes that are used to identify the term. The colour gradient reflects the pvalue significance (bright colour – low pvalue).

**Suppl. Fig. 18 Comparison with cell-cluster-defined gene sets.** A. Relationship between the differential expression and autocorrelation, shown using stacked bar charts. We used the neutrophil cluster, and calculated the markers using the Wilcoxon rank-sum test. The average log-fold values were binned in intervals of 0.25. The markers were grouped in four categories: genes that belong in the Starlng modules 15 (orange) or 25 (blue), autocorrelated genes associated with other modules (green) and genes with Moran's I test below 0.1 (pink). The barplots indicate the proportion of the gene groups; the labels show the size of each category. The solid line illustrates the percentage of marker genes assigned to the target modules with respect to the log-fold change value. B. Violin plots showing the distribution of the log-fold change (left) and expression variability (right) for each of the four gene categories. Set sizes (n) are indicated below each violin plot.

**Suppl. Fig. 19** **Robustness assessment of Starlng** The stability of the Starlng pipeline was evaluated using three experiments. The comparison is performed for each number of clusters that was obtained during the run (x-axis). A. Boxplot distribution of the Element-Centric Similarity (ECS) score between ground truth stable clusters and modules that are generated using a fraction of cells. B. Boxplot distribution of the ECS score calculated between ground truth stable clusters and modules that are generated after Gaussian-shaped noise (centred at 0 and variable standard deviation) is added to the normalised matrix. C. Boxplot distribution of the Element-Centric Consistency (ECC) score calculated on the list of partitions with the same number of clusters but generated with a different random seed. The assessment is performed both in the subsampling (left panel) and noise adding (right panel) experiments.

**Suppl. Fig. 20** **Sensitivity and stability analyses of pseudotime inference and module classification.** A. Scatterplots comparing cell-level pseudotime estimates before and after increasing the number of trajectory graph nodes, from 5 to 10 (left) and from 90 to 100 (right), representing the lower and upper ends of the range examined. The blue line shows the fitted regression line. B, C. Agreement between pseudotime vectors obtained using consecutive numbers of graph nodes (B) or graph iterations (C), assessed using the metrics indicated in the legend. EMD values are normalised; their maximum values are 0.21 in B and 0.02 in C. D. UMAP visualisation of candidate trajectory roots. Nodes are coloured by mean PCA distance within a pseudotime neighbourhood and sized according to the range of pseudotime values generated. E. Numbers of modules classified as filtered, redundant or outlier across values of the pseudotime IQR proportion used to define outlier behaviour. F. Median within-module UMAP distance for each module category across the tested pseudotime IQR proportions. G. Median within-module UMAP distance for each module category across the tested overlap thresholds used to classify redundant modules as filtered.

References

Aibar S *et al.* SCENIC: single-cell regulatory network inference and clustering. *Nature Methods* 2017;**14**:1083–6.

Andrews GK. Cellular zinc sensors: MTF-1 regulation of gene expression. *Biometals* 2001;**14**:223–37.

Arrojo e Drigo R *et al.* Structural basis for delta cell paracrine regulation in pancreatic islets. *Nature Communications* 2019;**10**:3700.

Ashburner M *et al.* Gene Ontology: tool for the unification of biology. *Nature Genetics* 2000;**25**:25–9.

Blondel VD *et al.* Fast unfolding of communities in large networks. *Journal of Statistical Mechanics: Theory and Experiment* 2008;**2008**:P10008.

Cao J *et al.* A human cell atlas of fetal gene expression. *Science* 2020;**370**:eaba7721.

Cao J *et al.* The single-cell transcriptional landscape of mammalian organogenesis. *Nature* 2019;**566**:496–502.

Cavalli M *et al.* A Multi-Omics Approach to Liver Diseases: Integration of Single Nuclei Transcriptomics with Proteomics and HiCap Bulk Data in Human Liver. *OMICS: A Journal of Integrative Biology* 2020;**24**:180–94.

Chang W *et al.*, *Shiny: Web Application Framework for R*, version 1.14.0 (21 June 2026).

Chevalley M *et al.* A large-scale benchmark for network inference from single-cell perturbation data. *Communications Biology* 2025;**8**:412.

Ching T *et al.*, *Qs2: Efficient Serialization of R Objects*, version 0.3.1 (21 Aug. 2026).

Craig-Schapiro R *et al.* Single-cell atlas of human pancreatic islet and acinar endothelial cells in health and diabetes. *Nature Communications* 2025;**16**:1338.

Csárdi G *et al.* igraph for R: R interface of the igraph library for graph theory and network analysis. 2026.

Cui EH *et al.* Single-cell generalized trend model (scGTM): a flexible and interpretable model of gene expression trend along cell pseudotime. *Bioinformatics* 2022;**38**:3927–34.

DeTomaso D, Yosef N. Hotspot identifies informative gene modules across modalities of single-cell genomics. *Cell Systems* 2021;**12**:446-56.e9.

Dibaeinia P, Sinha S. SERGIO: A Single-Cell Expression Simulator Guided by Gene Regulatory Networks. *Cell Systems* 2020;**11**:252-71.e11.

Ewing B *et al.*, *Leidenbase: R and C/C++ Wrappers to Run the Leiden Find_partition() Function*, version 0.1.37 (19 May 2026).

Fischer B, Smith M, Pau G, *Rhdf5* (Bioconductor, 2017).

Gao Q *et al.* SifiNet: a robust and accurate method to identify feature gene sets and annotate cells. *Nucleic Acids Research* 2024;**52**:e46.

Gates AJ *et al.* Element-centric clustering comparison unifies overlaps and hierarchy. *Scientific Reports* 2019;**9**:8574.

Geary RC. The Contiguity Ratio and Statistical Mapping. *The Incorporated Statistician* 1954;**5**:115–46.

Gribben C *et al.* Acquisition of epithelial plasticity in human chronic liver disease. *Nature* 2024;**630**:166–73.

Gu Z, Eils R, Schlesner M. Complex heatmaps reveal patterns and correlations in multidimensional genomic data. *Bioinformatics* 2016;**32**:2847–9.

Han Y-H *et al.* A maresin 1/ROR**α**/12-lipoxygenase autoregulatory circuit prevents inflammation and progression of nonalcoholic steatohepatitis. *The Journal of Clinical Investigation* 2019;**129**:1684–98.

Hao Y *et al.* Dictionary learning for integrative, multimodal and scalable single-cell analysis. *Nature Biotechnology* 2024;**42**:293–304.

Hartl CL *et al.* Coexpression network architecture reveals the brain-wide and multiregional basis of disease susceptibility. *Nature Neuroscience* 2021;**24**:1313–23.

Huynh-Thu VA *et al.* Inferring Regulatory Networks from Expression Data Using Tree-Based Methods. *PLOS ONE* 2010;**5**:e12776.

Jaitin DA *et al.* Lipid-Associated Macrophages Control Metabolic Homeostasis in a Trem2-Dependent Manner. *Cell* 2019;**178**:686-98.e14.

Jefferis G *et al.*, *RANN: Fast Nearest Neighbour Search (Wraps ANN Library) Using L2 Metric*, version 2.6.3 (24 Aug. 2026).

Jia Y, Wu H, Ding J, ‘scGeneRythm: Using Neural Networks and Fourier Transformation to Cluster Genes by Time-Frequency Patterns in Single-Cell Data’, preprint, bioRxiv, 27 Nov. 2023, 2023.11.26.568761.

Kang Y, Thieffry D, Cantini L. Evaluating the Reproducibility of Single-Cell Gene Regulatory Network Inference Algorithms. *Frontiers in Genetics* 2021;**12**.

Kiselev VY, Andrews TS, Hemberg M. Challenges in unsupervised clustering of single-cell RNA-seq data. *Nature Reviews Genetics* 2019;**20**:273–82.

Kolberg L *et al.* gprofiler2 -- an R package for gene list functional enrichment analysis and namespace conversion toolset g:Profiler. *F1000Research* 2020;**9**:ELIXIR-709.

Lähnemann D *et al.* Eleven grand challenges in single-cell data science. *Genome Biology* 2020;**21**:31.

Lange M *et al.* CellRank for directed single-cell fate mapping. *Nature Methods* 2022;**19**:159–70.

Langfelder P *et al.* Is My Network Module Preserved and Reproducible? *PLOS Computational Biology* 2011;**7**:e1001057.

Langfelder P, Horvath S. WGCNA: an R package for weighted correlation network analysis. *BMC Bioinformatics* 2008;**9**:559.

Langfelder P, Zhang B, Horvath S. Defining clusters from a hierarchical cluster tree: the Dynamic Tree Cut package for R. *Bioinformatics* 2008;**24**:719–20.

Lee JS *et al.* Transcriptional ontogeny of the developing liver. *BMC Genomics* 2012;**13**:33.

Levine JH *et al.* Data-Driven Phenotypic Dissection of AML Reveals Progenitor-like Cells that Correlate with Prognosis. *Cell* 2015;**162**:184–97.

Liu J *et al.* Wnt/β-catenin signalling: function, biological mechanisms, and therapeutic opportunities. *Signal Transduction and Targeted Therapy* 2022;**7**:3.

Martin OP *et al.* Single-cell atlas of human liver and blood immune cells across fatty liver disease stages reveals distinct signatures linked to liver dysfunction and fibrogenesis. *Nature Immunology* 2025;**26**:1596–611.

Matys V *et al.* TRANSFAC® and its module TRANSCompel®: transcriptional gene regulation in eukaryotes. *Nucleic Acids Research* 2006;**34**:D108–10.

McInnes L *et al.* UMAP: Uniform Manifold Approximation and Projection. *Journal of Open Source Software* 2018;**3**:861.

Morabito S *et al.* hdWGCNA identifies co-expression networks in high-dimensional transcriptomics data. *Cell Reports Methods* 2023;**3**.

Moran PAP. A test for the serial independence of residuals. *Biometrika* 1950;**37**:178–81.

Moutsopoulos I *et al.* noisyR: enhancing biological signal in sequencing datasets by characterizing random technical noise. *Nucleic Acids Research* 2021;**49**:e83.

Pliner HA, Shendure J, Trapnell C. Supervised classification enables rapid annotation of cell atlases. *Nature Methods* 2019;**16**:983–6.

Radi SH *et al.* HNF4α isoforms: the fraternal twin master regulators of liver function. *Frontiers in Endocrinology* 2023;**14**.

Regev A *et al.* The Human Cell Atlas. *eLife* 2017;**6**:e27041.

Reichardt J, Bornholdt S. Statistical mechanics of community detection. *Physical Review E* 2006;**74**:016110.

Risso D *et al.* A general and flexible method for signal extraction from single-cell RNA-seq data. *Nature Communications* 2018;**9**:284.

Rorsman P, Ashcroft FM. Pancreatic β-Cell Electrical Activity and Insulin Secretion: Of Mice and Men. *Physiological Reviews* 2018;**98**:117–214.

Rubner Y, Tomasi C, Guibas LJ, ‘A Metric for Distributions with Applications to Image Databases’, *Sixth International Conference on Computer Vision* (Jan. 1998), 59–66.

Saelens W *et al.* A comparison of single-cell trajectory inference methods. *Nature Biotechnology* 2019;**37**:547–54.

Saelens W, Cannoodt R, Saeys Y. A comprehensive evaluation of module detection methods for gene expression data. *Nature Communications* 2018;**9**:1090.

Shahsavari A, Munteanu A, Mohorianu I, ‘ClustAssess: Tools for Assessing the Robustness of Single-Cell Clustering’, preprint, bioRxiv, 2 Feb. 2022, 2022.01.31.478592.

Song D, Li JJ. PseudotimeDE: inference of differential gene expression along cell pseudotime with well-calibrated p-values from single-cell RNA sequencing data. *Genome Biology* 2021;**22**:124.

Song Q, Wang J, Bar-Joseph Z. scSTEM: clustering pseudotime ordered single-cell data. *Genome Biology* 2022;**23**:150.

Stock M *et al.* Leveraging prior knowledge to infer gene regulatory networks from single-cell RNA-sequencing data. *Molecular Systems Biology* 2025;**21**:214–30.

Tirosh I *et al.* Dissecting the multicellular ecosystem of metastatic melanoma by single-cell RNA-seq. *Science* 2016;**352**:189–96.

To JC *et al.* ZBTB20 regulates WNT/CTNNB1 signalling pathway by suppressing PPARG during hepatocellular carcinoma tumourigenesis. *JHEP Reports* 2021;**3**.

Traag VA, Waltman L, Van Eck NJ. From Louvain to Leiden: guaranteeing well-connected communities. *Scientific Reports* 2019;**9**:5233.

Trapnell C. Defining cell types and states with single-cell genomics. *Genome Research* 2015;**25**:1491–8.

Wickham H. *ggplot2: Elegant Graphics for Data Analysis*. Cham: Springer International Publishing, 2016.

Wolf FA *et al.* PAGA: graph abstraction reconciles clustering with trajectory inference through a topology preserving map of single cells. *Genome Biology* 2019;**20**:59.

Xie Y *et al.*, *DT: A Wrapper of the JavaScript Library ‘DataTables’*, version 0.34.0 (2 Sept. 2025).

Xu Q *et al.* scInTime: A Computational Method Leveraging Single-Cell Trajectory and Gene Regulatory Networks to Identify Master Regulators of Cellular Differentiation. *Genes* 2022;**13**:371.
